# Supplementary material for: Metabolic Reprograming Via Silencing of Mitochondrial VDAC1 Expression Encourages Differentiation of Cancer Cells
Source: Mol Ther Nucleic Acids. 2019 May 18;17:24–37. doi: 10.1016/j.omtn.2019.05.003 (PMC6562189; doi:10.1016/j.omtn.2019.05.003)
Supplement: Document S1. Figures S1 and S2 and Tables S1–S4 [file mmc1.pdf]

OMTN, Volume 17

Supplemental Information

Metabolic Reprograming Via Silencing of Mitochondrial VDAC1 Expression Encourages Differentiation of Cancer Cells

Tasleem Arif, Zohar Amsalem, and Varda Shoshan-Barmatz

Supplementary Data

Metabolic Reprograming via Silencing of Mitochondrial VDAC1 Expression Encourages Differentiation of Cancer Cells

Tasleem Arif,* Zohar Amsalem* and Varda Shoshan-Barmatz#

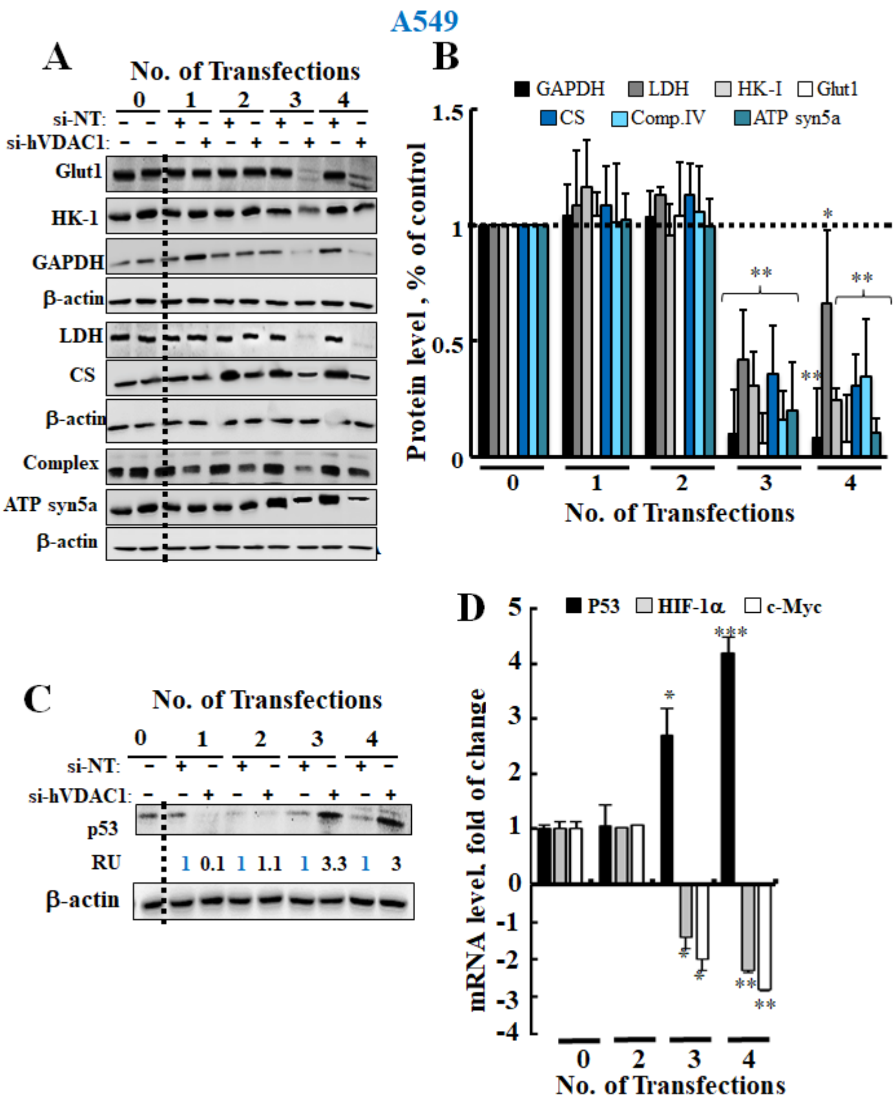

Figure S1. si-hVDAC1 Treatment of A549 Cells Reprogrammed Metabolism and Altered TF Expression Following the Third and Fourth Transfections

Immunoblotting (**A**) and quantitative analysis (**B**) of Glut-1, HK-I, GAPDH, LDH-A, CS, complex IVc and ATP 5a synthase in si-NT- or si-hVDAC1-treated A549 cells following the indicted transfection. β-actin served as an internal loading control. Results reflect the mean ± SEM. The dashed line indicates the level in the controls. **(C)** Immunoblot analysis of p53 levels in si-NT- and si-hVDAC1-treated A549 cells following the indicated transfection. RU represents the relative units (RU) of expression of the indicated protein in si-hVDAC1-treated A549 cells relative to si-NT-treated A549 cells. **(D)** TF mRNA expression levels as analyzed using qRT-PCR with specific primers. The levels of p53 (black bar), HIF-1α (gray bar) and c-Myc (white bar) in si-hVDAC1-treated A549 cells relative to those in si-NT-treated A549 cells following the indicated transfection were determined. Results reflect the mean ± SEM, p*≤0.05; p**≤0.01; p***≤0.001.

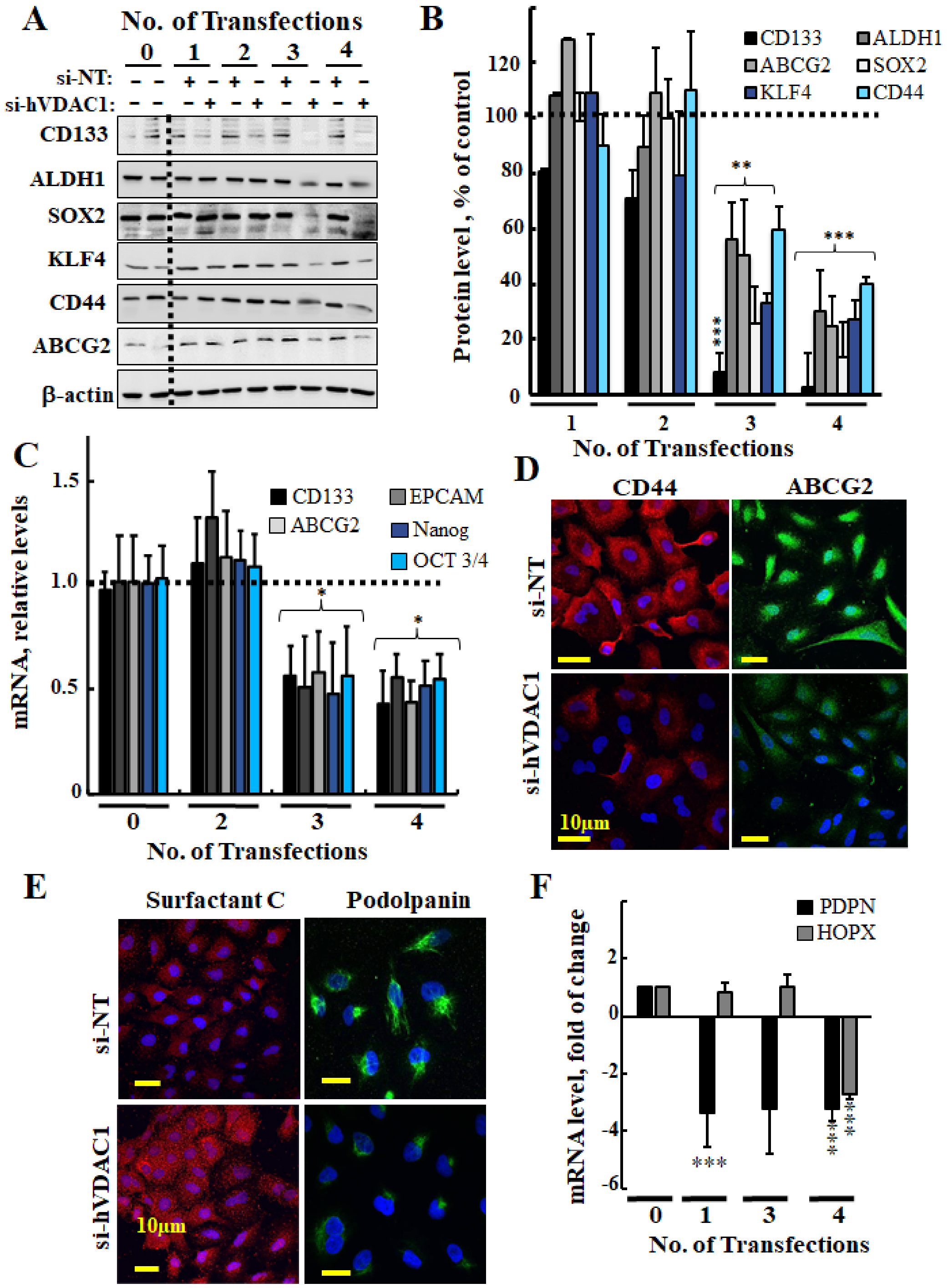

Figure S2. si-hVDAC1 Treatment of A549 Cells Markedly Reduced the Expression of CSC Markers and Proteins Associated with Differentiation Following the Third and Fourth Transfections

Immunoblotting (**A**) and quantitative analysis (**B**) of selected CSC markers (CD133, ALDH1, SOX2, KLF4, CD44 and ABCG2) in si-NT- and si-hVDAC1-treated A549 cells at the indicated transfection, using specific antibodies. β-actin served as an internal loading control. The dashed line indicates the level in the controls. (**C**) q-RT-PCR of mRNA levels of CSC markers (CD133, EPCAM, ABCG2, Nango and Oct3/4) in A549 cells treated with si-NT or si-hVDAC1 for the indicated number of transfections, and presented relative to their levels in si-NT-treated cells. Results reflect the mean ± SEM, p: $*\leq 0.05$. **(D,E)** IF staining of si-NT- and si-hVDAC-treated cells following the fourth transfection stained for CD44 and ABCG2 (**D**) and surfactant C, and for podoplanin after third transfection (**E**), as visualized by confocal microscopy. **(F)** qRT-PCR analysis of mRNA levels of podoplanin (PDPN, black bar), and homedomain only protein x (HOPX, gray bar) in si-hVDAC1-treated A549 cells, relative to those in si-NT-treated A549 cells, following the indicated transfection. Results reflect the means ± SEM; $p^{**}\leq 0.01$; $p^{***}\leq 0.001$.

Table S1. Summary of the Genes Expression Levels Associated with Metabolism, Stem Cells, Differentiation and Transcription Factors as a Function of Number of Transfections of MDA-MB231 Cells with si-hVDAC1, Relative to Transfection with si-NT; q-PCR Results

	Fold change si-NT/si-hVDAC1			
**Transfection No**	**0**	**2**	**3**	**4**
**Gene**				
**Metabolism**				
Glut1	1.00 ± 0.15	-0.56 ± 0.61	-1.05 ± 0.51	-2.74 ±0.34
HK-1	1.00 ± 0.36	-1.07 ± 0.06	-3.84 ± 0.14	-9.86 ± 0.15
GAPDH	1.00 ± 0.08	-1.83 ± 0.29	-3.66 ± 0.26	-10.59 ± 0.05
LDH	1.00 ± 0.07	-0.67 ± 0.10	-1.34 ± 0.08	-2.72 ± 0.22
CS	1.00 ± 0.22	-0.78 ± 0.13	-1.01 ± 0.33	-2.56 ± 0.35
ATP syn5a	1.00 ± 0.10	-1.4 ± 0.09	-1.81 ± 0.0.4	-6.35 ± 0.04
VDAC1	1.00 ± 0.00	-3.49 ± 0.15	-4.72 ± 0.08	-4.76 ± 0.07
VDAC2	1.00 ± 0.10	-1.15 ± 0.29	-1.04 ± 0.26	-0.81 ± 0.05
VDAC3	1.00 ± 0.00	-0.94 ± 0.33	-0.913 ± 0.14	-0.90 ± 0.15
**Differentiation**				
PR	1.00	1.05±0.24	1.51±0.41	3.051±0.70
PRL	1.00	1.94±0.35	2.55±0.21	1.95±0.17
ESR1	1.00	7.98±1.68	7.61±1.37	5.34±1.73
**TF**				
p53	1.00 ± 0.03	1.12 ± 0.01	6.29 ± 0.38	6.00 ± 0.36
c-MYC	1.00 ± 0.05	-1.06 ± 0.08	-3.13 ± 0.14	-2.64 ± 0.04
HIF1-alpha	1.04 ± 0.05	-1.06 ± 0.09	-1.25 ± 0.05	-4.51± 0.04

Table S2. Summary of The Expression Levels of Genes Associated with Metabolism, Stem Cells, Differentiation and Transcription Factors as a Function of The Number of Transfections of A549 Cells with si-hVDAC1 Relative to Transfection with si-NT; q-PCR Results

	**Fold change si-NT/siVDAC1**			
**Transfection No:**	**0**	**2**	**3**	**4**
**Gene**				
**Metabolism**				
Glut1	1.00 ± 0.1	-1.37 ± 0.12	-2.60 ± 0.21	-3.09 ± 0.45
HK-1	1.00 ± 0.01	-2.37 ± 0.23	-1.03 ± 0.09	-13.09 ± 1.97
LDH	1.00 ± 0.07	-0.67 ± 0.10	-1.34 ± 0.08	-2.72 ± 0.22
CS	1.00 ± 0.13	-0.93 ± 0.08	-1.9 ± 0.21	-4.12 ± 0.67
ATP syn5a	1.00 ± 0.01	-1.00 ± 0.10	-2.02 ± 0.26	-3.99 ± 0.54
VDAC1	1.00 ± 0.05	-2.84 ± 0.05	-8.46 ± 0.05	-6.76 ± 0.01
VDAC2	1.00 ± 0.02	-1.19 ± 0.01	-1.17 ± 0.10	-1.20 ± 0.05
VDAC3	1.00 ± 0.24	-1.11 ± 0.30	- 0.96 ± 0.14	-0.74 ± 0.05
**Stem Cell**				
CD133	1.00 ± 0.02	-1.13 ± 0.22	-1.71 ± 0.13	-2.24 ± 0.15
EPCAM	1.00 ± 0.05	-0.76 ± 0.15	-2.00 ± 0.15	-1.89 ± 0.12
ABCG2	1.00 ± 0.09	-0.98 ± 0.22	-1.74 ± 0.13	-2.31 ± 0.15
NANOG	1.00 ± 0.01	-0.89 ± 0.20	-2.12 ± 0.25	-1.99 ± 0.24
OCT3/4	1.00 ± 0.05	-0.95 ± 0.10	-1.83 ± 0.11	-1.88 ± 0.11
**Differentiation**				
PDPN	1.00 ± 0.01	0.84 ± 0.32	1.02 ± 0.42	-2.71 ± 0.16
HOPX	1.00 ± 0.01	-3.36 ±1.63	-3.23 ± 1.58	-3.24 ± 0.40
**TF**				
p53	1.00 ± 0.05	1.05 ± 0.20	2.69 ± 0.05	4.19 ± 0.05
c-MYC	1.00 ± 0.05	-1.02 ± 0.24	-1.96 ± 0.30	-2.84 ± 0.05
HIF1-alpha	1.04 ± 0.05	-1.06 ± 0.50	-1.40 ± 0.30	-2.37 ± 0.05

Table S3. Antibodies Used in This Study

Antibodies against the indicated protein, their catalogue number, source, and the dilutions used in immunoblot and immunofluorescence experiments are presented.

Antibodies	Source and Cat. No.	Dilution		
		IHC	WB	IF
Mouse monoclonal anti-β-actin	Millipore, Billerica, MA, MAB1501	-	1:10000	-
Rabbit monoclonal anti-VDAC1	Abcam, Cambridge, UK , ab154856	-	1:5000	-
Rabbit monoclonal cytochrome c oxidase subunit IVc	Abcam, Cambridge, UK, ab150422	-	1:2000	-
Rabbit polyclonal anti -NGFR	Santa Cruz Biotechnology, Inc. Dallas, TX, sc-8317	-	1:1000	-
Rabbit monoclonal anti-ALDH1	Abcam, Cambridge, UK , ab52492	-	1:2000	-
Rabbit polyclonal anti-BCL-xL	Abcam, Cambridge, UK , ab98143	-	1:4000	-
Mouse monoclonal anti-Ezrin	Santa Cruz Biotechnology, Inc. Dallas, TX, sc-58758	-	-	1:500
Mouse monoclonal anti-Podoplanin	Abcam, Cambridge, UK, ab10288	-	-	1:500
Rabbit monoclonal anti-Glut1	Abcam, Cambridge, UK ab40084	-	1: 1500	-
Mouse monoclonal anti-GAPDH	Abcam, Cambridge, UK, ab9484	-	1:1000	-
Rabbit polyclonal anti-citrate synthase	Abcam, Cambridge, UK ab96600	-	1:4000	-
Mouse monoclonal anti-Cytochrome *c*	B.D Bioscience, San Jose, CA, Cat. No 556432	-	1:2000	-
Mouse monoclonal anti-ATP5a	Abcam, Cambridge, UK, ab14748	-	1:1000	-
Mouse monoclonal anti-HK-I	Abcam, Cambridge, UK ab105213	-	1:2000	-
Goat polyclonal anti-LDH-A	Santa Cruz Biotechnology, Inc. Dallas, TX, sc-27230	-	1:1500	-
Rabbit monoclonal anti-LDH	Epitomics, Cambridge, UK, 1980-1	-	1:1000	-
Rabbit polyclonal anti-Nestin	Millipore, Billerica, MA, MAB353	-	1:1000	1:500
Goat polyclonal anti-Sox2	Santa Cruz Biotechnology, Inc. Dallas, TX, sc-17320	-	1:1500	1:300
Rabbit polyclonal anti-KLF4	IMGENX Littleton, USA, IMG-6081-A	-	1:1000	-
Mouse monoclonal anti-CD133	Miltenyi Biotec GmbH , AC133	-	1:1500	-
Rabbit polyclonal anti-Prosurfactant protein C	Abcam, ab90716	-	-	1:250
Mouse monoclonal anti-β III tubulin	Abcam, Cambridge, UK ab7751	-	1:5000	1:200
Mouse monoclonal anti-GFAP	Santa Cruz Biotechnology, Inc. Dallas, TX, sc-33673	-	1:5000	1:150
Rabbit polyclonal anti-AMPK-p	Abcam, Cambridge, UK, ab23875	-	1:5000	-
Rabbit polyclonal anti-pS6	Cell Signaling Technology, Danvers, USA, cat: 2215	-	1:1000	-
Mouse monoclonal anti-P53	Santa Cruz Biotechnology, Inc. ,Dallas, TX, sc-126	-	1:5000	-

Mouse monoclonal anti-ABCG2	GeneTex, Irvine, CA, cat: GTX60447	-	1:2000	1:500
Rabbit polyclonal anti-CD44	Abcam, Cambridge, UK, ab157107	-	1:3000	1:1000
Mouse monoclonal anti-ERB2/HER-2	Biolegend, San Diego, CA, cat: 324401	-	-	1:500
Alexa Fluor 488 anti-mouse/human CD44	Biolegend, San Diego, CA, cat: 103015	-	-	1:500
Goat anti-Rabbit IgG H&L (Alexa Fluor 555)	Abcam, Cambridge, UK, ab150086	-	-	1:500
Cow polyclonal anti-goat IgG FITC	Santa Cruz Biotechnology, Inc. Dallas, TX, sc-2348	-	-	1:500
Mouse monoclonal anti-CD24	Biolegend, San Diego, CA, cat: 311101	-	-	1:500

Table S4: Real-Time PCR Primers Used in This Study

The genes examined and the forward and reverse sequences of the primers used are indicated.

Gene	Primer sequences
ß-Actin	Forward 5’-ACTCTTCCAGCCTTCCTTCC-3’<br>Reverse 5’- TGTTGGCGTACAGGTCTTTG-3’
VDAC1	Forward 5’- AATGACGGGACAGAGTTTGG<br>Reverse 5’- AGCGCGTGTTACTGTTTCCT
VDAC2	Forward 5’- GGGGACTTCCAGCTACACAC-3’<br>Reverse 5’- CCTGATGTCCAAGCAAGGTT-3’
VDAC3	Forward 5’- AATAATGCCAGCCTGATTGG-3’<br>Reverse 5’- CTTGTGACCTCCTGCACTGA-3’
GLUT1	Forward 5’- GGCCATCTTTTCTGTTGGGG-3’<br>Reverse 5’- TCAGCATTGAATTCCGCCG-3’
GAPDH	Forward 5’- TGGAAGGACTCATGACCACA-3’<br>Reverse 5’- ATGATGTTCTGGAGAGCCCC-3’
LDH-A	Forward 5’-GCAGGTGGTTGAGAGTGCTT-3’<br>Reverse 5’- GCACCCGCCTAAGATTCTTC-3’
CS	Forward 5’- AGGAACAGGTATCTTGGCTCT-3’<br>Reverse 5’- GGGGTGTAGATTGGTGGGAA-3’
ATP syn5a	Forward 5’-TCAGTCTACGCCGCACTTAC-3’<br>Reverse 5’- GACATCTCAGCAGTCCCACA-3’
HK-I	Forward 5’-GTCTCAGTCCAGCACGTTTG-3’<br>Reverse 5’- GAAACGCCGGGAATACTGTG-3’
KLF4	Forward 5’-TGCCCCGAATAACCGCTG-3’<br>Reverse 5’-CGTTGAACTCCTCGGTCTCT-3’
ABCG2	Forward 5’- CACCTTATTGGCCTCAGGAA-3’<br>Reverse 5’- GAAACACTGGTTGGTCGTCA -3’
CD133	Forward 5′-TGGGCTTGTCATAACAGGAT-3′<br>Reverse 5′-TTGCGGTAAAACTGGCTAAG-3′
EPCAM	Forward 5′-CTGGCCGTAAACTGCTTTGT-3′<br>Reverse 5′-TCCCAAGTTTTGAGCCATTC-3′
Nestin	Forward 5’- GAAACAGCCATAGAGGGCAAA-3’<br>Reverse 5’- TGGTTTTCCAGAGTCTTCAGTGA-3’

Nanog	Forward 5'- TGGGATTTACAGGCGTGAGCCAC-3'<br>Reverse 5'- AAGCAAAGCCTCCCAATCCCAAAC-3'
SOX2	Forward 5'- CCATGCAGGTTGACACCGTTG -3'<br>Reverse 5'-TCGGCAGACTGATTCAAATAA-3'
TUBB3	Forward 5'-CTCAGGGGCCTTTGGACATC-3'<br>Reverse 5'-CAGGCAGTCGCAGTTTTCAC-3'
MAP2	Forward 5'-TCCAAAATCGGATCAACAGAC-3'<br>Reverse 5'- AGAGCCACATTTGGATGTCAC-3'
GFAP	Forward 5'- AAGCTCCAGGATGAAACCAAC-3'<br>Reverse 5'- AGCGACTCAATCTTCCTCTCC-3'
c-Myc	Forward 5'-GTAGTGGAAAACCAGCAGCC-3'<br>Reverse 5'- CCTCCTCGTCGCAGTAGAAA-3'
p53	Forward 5′-AGGTTGGCTCTGACTGTACC-3′<br>Reverse 5′-AAAGCTGTTCCGTCCCAGTA-3
Hif-1 α	Forward 5'-CTGACCCTGCACTCAATCAA-3'<br>Reverse 5'- TCCATCGGAAGGACTAGGTG-3'
Cytochrome c	Forward 5′-TTTGGATCCAATGGGTGATGTTGAG-3<br>Reverse 5'TTGAATTCCTCATTAGTAGCTTTTTTGAG-3
HOPX	Forward 5'- TTAAGCAGGCTGGCATCAG-3'<br>Reverse 5'- TGCTCCGCTAGACCCTTCT-3'
Podoplanin	Forward 5'- ATCTGCCAACTTCAGAAAGCA-3'<br>Reverse 5'- TTGTCTGTGTGTCTCCATCCA-3'
PRLR	Forward 5'-AATCTTGGCAGAGGCAGAAA-3'<br>Reverse 5'-TTTGGAGCTATTCCCATTGC-3'
PR	Forward 5'- GTCTACCCGCCCTATCTCAAC-3'<br>Reverse 5'- ACCATAATGACAGCCTGATGC-3'
ESRI	Forward 5'-TGGAGATCTTCGACATGCTG -3'<br>Reverse 5'- TCCAGAGACTTCAGGGTGCT -3'
