# Supplementary material for: Metabolic Reprograming Via Silencing of Mitochondrial VDAC1 Expression Encourages Differentiation of Cancer Cells
Source: Mol Ther Nucleic Acids. 2019 May 18;17:24–37. doi: 10.1016/j.omtn.2019.05.003 (PMC6562189; doi:10.1016/j.omtn.2019.05.003)
Supplement: Document S2. Article plus Supplemental Information [file mmc2.pdf]

Molecular Therapy
Nucleic Acids
Original Article

AMERICAN SOCIETY of GENE & CELL THERAPY

Metabolic Reprograming Via Silencing of Mitochondrial VDAC1 Expression Encourages Differentiation of Cancer Cells

Tasleem Arif,[1,2,3] Zohar Amsalem,[1,3] and Varda Shoshan-Barmatz[1]

[1]Department of Life Sciences and the National Institute for Biotechnology in the Negev, Ben-Gurion University of the Negev, Beer-Sheva 84105, Israel

The mitochondrial gatekeeper voltage-dependent anion channel 1 (VDAC1) controls metabolic and energy cross-talk between mitochondria and the rest of the cell and is involved in mitochondria-mediated apoptosis. Here, we compared the effects of downregulated VDAC1 expression in the U-87MG glioblastoma, MDA-MB-231 triple-negative breast cancer (TNBC), and A549 lung cancer cell lines, using small interfering RNA (siRNA) specific to human VDAC1 (si-hVDAC1). The cells were subjected to si-hVDAC1 (50 nM) treatment for 5–20 days. Although VDAC1 silencing occurred within a day, the cells underwent reprograming with respect to rewiring metabolism, elimination of cancer stem cells (CSCs), and alteration of transcription factor (TF) expression and proteins associated with differentiation, with maximal changes being observed after 3 weeks of silencing VDAC1 expression. The differentiation into fewer tumorigenic cells may be associated with the elimination of CSCs. These alterations are interconnected, as protein up- or downregulation occurred simultaneously, starting 15–20 days after VDAC1 levels were first decreased. Moreover, the VDAC1 depletion-mediated effects on a network of key regulators of cell metabolism, CSCs, TFs, and other factors leading to differentiation are coordinated and are common to the glioblastoma multiforme (GBM) and lung and breast cancer cell lines, despite differing in origin and carried mutations. Thus, our study showed that VDAC1 depletion triggers reprograming of malignant cancer cells into terminally differentiated cells and that this may be a promising therapeutic approach for various cancers.

INTRODUCTION

Cancer cells undergo reprograming of metabolism and develop survival strategies recognized as hallmarks of cancer. Cancer cells exhibit significant metabolic alterations related to critical nutrients and substrates, such as glucose, glutamine, and oxygen, and show plasticity of the metabolic machinery.[1,2] Accumulating evidence indicates that cancer cells use a combination of both glycolysis and oxidative phosphorylation (OXPHOS) to produce ATP, with the ratio depending on the prevalent normoxic or hypoxic environmental conditions.[3–5]

The mitochondrial protein voltage-dependent anion channel 1 (VDAC1) mediates the fluxes of ions, nucleotides, and other metabolites (e.g., pyruvate, malate, succinate, and NADH/NAD$^+$), as well as hemes, fatty acids, and cholesterol across the outer mitochondrial membrane (OMM).[6] VDAC1 at the OMM interacts with proteins that mediate and regulate the integration of signals generated elsewhere in the cell.[6–8] VDAC1 also contributes to cancer cell metabolism via the binding and channelling of mitochondrial ATP directly to hexokinase (HK), the first enzyme in glycolysis, also highly expressed in various cancers.[5] This results in mitochondria regulating glycolytic flux with the TCA cycle and ATP synthase to fulfill the requirements of the tumor for metabolites or metabolic precursors. VDAC1 is highly expressed in different tumors,[7,9] pointing to its pivotal role in regulating cancer cell energy and metabolism.

As cellular metabolic and energy reprograming of cancer cells are essential for tumor progression, blocking energy production and the assembly of building blocks in the cancer cell is a promising anti-cancer strategy. There are currently drugs that target a single metabolic enzyme, but none that target global metabolism. VDAC1, which governs metabolic cross-talk between the mitochondria and other cell compartments and is crucial, not only for metabolic functions of the mitochondria, but also for glycolysis and other cellular metabolic processes, may represent such a target.

Using human-specific small interfering-hVDAC1 (si-hVDAC1) to silence the expression of VDAC1 reduces cellular ATP levels and cell growth,[10] and inhibits tumor growth in lung cancer[9] and glioblastoma multiforme (GBM).[11] Upon treatment of GBM tumors with si-hVDAC1, the residual tumor shows reversed oncogenic properties, such as rewired metabolism and reduced angiogenesis, invasiveness, and stemness, leading to differentiation into neuron- and astrocyte-like cells.[11] Many factors control cell differentiation, including cell

Received 28 September 2018; accepted 9 May 2019;
https://doi.org/10.1016/j.omtn.2019.05.003.

[2]Present address: Department of Cell, Developmental and Regenerative Biology, Icahn School of Medicine at Mount Sinai, New York, NY- 10029, USA

[3]These authors contributed equally to this work.

Correspondence: Varda Shoshan-Barmatz, Department of Life Sciences and the National Institute for Biotechnology in the Negev, Ben-Gurion University of the Negev, Beer-Sheva 84105, Israel.
E-mail: vardasb@bgu.ac.il

 © 2019 The Authors.
This is an open access article under the CC BY license (http://creativecommons.org/licenses/by/4.0/).

metabolism. In addition, cancer cells do not undergo differentiation as part of their maturation, but instead, continuously proliferate.

Accumulated evidence supports the cancer stem cell (CSC) hypothesis, which suggests that a sub-population of malignant cells exhibits the stem cell properties of self-renewal and differentiation[12] and is resistant to conventional cytotoxic/anti-proliferative therapies.[12,13] CSC sub-populations have been identified in nearly all human malignancies. In GBM, CD133, SSEA1, CD49f, Musashi-1, and nestin are considered to be glioma stem cell (GSC) markers.[12,14] We demonstrated a global change in tumor hallmarks upon silencing VDAC1 expression in GBM mouse models.[11]

Breast cancer is a heterogeneous disease with several subtypes, defined based on the presence or absence of estrogen receptors (ERs), progesterone receptors (PRs), and epidermal growth factor receptor-2, the receptor tyrosine-protein kinase erbB-2 (ERBB2/Her2). The majority (>60%) of breast cancers are ER-positive, whereas about 20% are defined as triple-negative breast cancer (TNBC), negative for ER, PR, and HER2 expression.[15] There are only limited therapies for TNBC.[16] Breast cancer stem-cell-specific markers include BMI-1, CD24, CD44, CD49f, aldehyde dehydrogenase (ALDHA1), and EpCAM. $CD242^{low}/CD44^{+}$/ALDH have been proposed as having high tumorigenic potential in both human primary tumors and cell lines.[17–19] In human lung cancer, CSCs have also been described,[20–23] with markers including the metabolic marker alcohol aldehyde dehydrogenase1 (ALDH1) and the cell surface markers CD133 and CD166.[24]

In this study, we asked whether silencing VDAC1 expression in cultured cancer cells would result in tumors showing rewired metabolism, altered TF levels, elimination of CSCs, and induction of differentiation. This would allow us to evaluate the contribution of the tumor microenvironment to cancer cell reprograming. Accordingly, the U-87MG glioblastoma, MDA-MB-231 TNBC, and A549 lung cancer cell lines were subjected to si-hVDAC1 treatment for 5–20 days. Although VDAC1 silencing occurred within the first day, cell reprograming, leading to rewired metabolism, inhibited cell proliferation and stemness, altered expression of TFs, and induction of differentiation all developed with time, with maximal changes seen after 3 weeks of VDAC1 silencing. The results demonstrate that the interplay between metabolism and oncogenic signaling networks is common to GBM and lung and breast cancers, despite differing in origin and carried mutations. Our results suggest that depletion of VDAC1, serving as the bottleneck between mitochondrial and cellular metabolism, can be considered a novel strategy to target various cancers.

RESULTS

After our previous studies in a GBM mouse model demonstrated that si-hVDAC1 inhibits tumor growth, reprograms metabolism, inhibits stemness, and leads to cell differentiation,[11] we asked whether similar effects would be obtained in cells in culture without the influence of the tumor microenvironment and in other cancer cell lines differing in origin and mutations carried.

VDAC1 Depletion Inhibits Cancer Cell Growth and ATP Production and Alters the Expression of Metabolic Enzymes

We initially focused on U-87MG cells, as GBM is a heterogeneous disease with a complex tumor microenvironment comprising many layers of tumor cells and normal inflammatory cells. Within this heterogeneity, the bulk tumor, proliferating cells, and cancer stem cells are found.[25] Mutations in metabolic enzymes or signaling pathways directly regulating glucose and mitochondrial metabolism have been identified in GBM.[26–28]

Silencing of VDAC1 expression in U-87MG cells was carried out four times, every 5 days (Figure 1A), using a specific human (h)VDAC1 siRNA sequence (nt 238–256). At these times, VDAC1 expression levels were analyzed at the protein and mRNA levels (Figures 1B–1D). si-hVDAC1 markedly decreased VDAC1 protein levels (by 80%) from the first to the fourth transfections (Figure 1C). Similarly, mRNA levels were decreased by 65% following the first transfection and by 75% after the fourth transfection (Figure 1D). The si-hVDAC1 was specific to VDAC1 and had no effect on VDAC2 or -3 mRNA levels (Figure 1D). Non-targeted si-RNA (NT-siRNA) had no significant effect on VDAC1 levels (Figures 1B).

Cells expressing low VDAC1 levels possessed low membrane potential ($\Delta\Psi$) (Figure 1E), low cellular ATP levels (Figure 1F), and markedly decreased cell proliferation, as assayed by the sulforhodamine B (SRB) method (Figure 1G) or by observing the expression level of the cell proliferation factor Ki-67, as analyzed by real-time qPCR (Figure 1H).

Although VDAC1 levels were decreased after the first transfection with si-hVDAC1, only after the third and fourth transfections did si-hVDAC1-treated cells show marked decreases in the expression levels of all tested metabolism-related proteins. These included glucose transporter 1 (Glut-1), hexokinase (HK-I), glyceraldehyde dehydrogenase (GAPDH), and lactate dehydrogenase-A (LDH-A), as compared with their levels in the si-NT-treated cells (Figures 2A–2C). Expression levels of the Kreb's cycle enzyme citrate synthase (CS), mitochondrial electron transport complex IVc, and ATP synthase subunit 5a were also highly reduced in si-hVDAC1-treated cells after the third and fourth transfections, consistent with alterations in OXPHOS. Similar results were obtained at the mRNA level (Figures 2C and 2D). Glut-1 showed the highest decreases at both the mRNA and protein levels after the third transfection. Upregulated Glut-1 expression fosters enhanced aerobic glycolysis and is a common hallmark of many cancers.[2]

As cancer metabolism reprograming involves interconnected signaling cascades, as, for example, mediated by the AMP-activated protein kinase (AMPK), a central regulator of cellular metabolism, energy, and redox homeostasis under various metabolic stress conditions was proposed to possess both anti- and pro-tumorigenic properties.[29,30] High levels of the phosphorylated/activated AMPK (p-AMPK) protein were found in si-NT-treated cells that were highly decreased in cells transfected three and four times with si-hVDAC (Figures 2E and 2F). Thus, the decrease in the level of activated AMPK in si-hVDAC1-treated

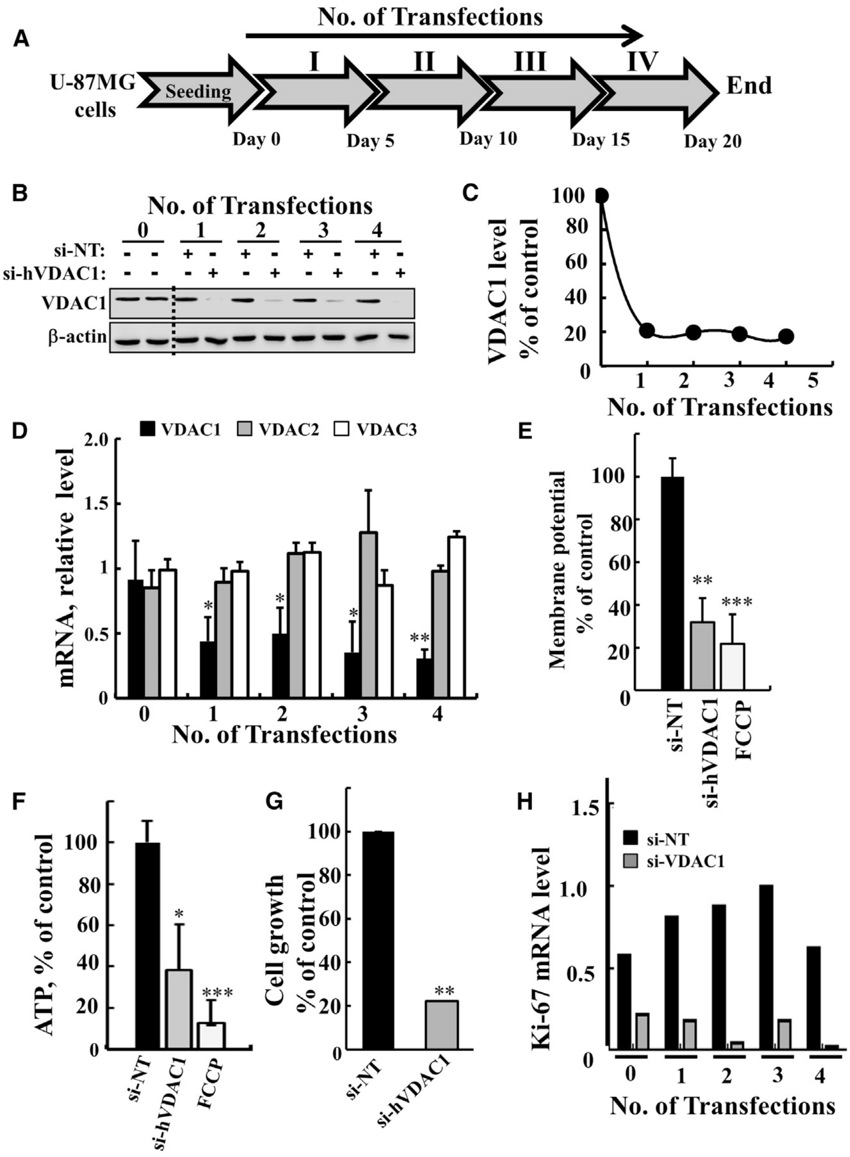

Figure 1. si-hVDAC1 Treatment Silences VDAC1 Expression, Reduces Energy Production, and Inhibits Cell Growth as Early as after the First Transfection

(A) Experimental protocol for si-hVDAC1 treatment of the U-87-MG cell line. Cells (150,000 cells/well in a 6-well plate) were transfected with 50 nM of non-targeting si-RNA (si-NT) or si-RNA against human hVDAC1 (si-hVDAC1) four times at 5-day intervals. (B and C) VDAC1 expression levels were analyzed after the indicated transfection by immunoblot (B) and quantitation (C). (D) mRNA expression levels for VDAC1 (black bar), VDAC2 (gray bar), and VDAC3 (white bar), as analyzed at the indicated transfection using real-time qPCR and specific primers. (E and F) Mitochondrial membrane potential ($\Delta\Psi$) (E) and ATP levels (F) were analyzed after the third transfection, as described in Materials and Methods. Cells were treated with si-NT (black bars), si-hVDAC1 (gray bars), or FCCP (20 μM) as a positive control (white bars). (G) Growth of cells transfected four times with si-NT (black bar) or si-hVDAC1 (gray bar) was assayed by using the SRB method. (H) Proliferation of cells transfected four times with si-NT (black bar) or si-hVDAC1 (gray bar) was assayed by using real-time qPCR analysis of the proliferation marker Ki-67. Results reflect the mean ± SEM; *$p \leq 0.05$; **$p \leq 0.01$; ***$p \leq 0.001$.

cells (Figures 2E and 2F) is consistent with this kinase's being a possible target for cancer prevention and treatment.[31]

Similar results were obtained with the phosphorylated form of ribosomal S6 (p-S6). S6 represents a mammalian target of rapamycin (mTORC1), a serine/threonine protein kinase that regulates cell growth and proliferation by integrating signals arising from growth factors, nutrients, and energy status.[32,33] Thus, the decrease in the level of phosphorylated S6 (Figures 2E and 2F) suggests that mTOR kinase activity was also reduced.

Cell Treatment with si-hVDAC1 Eliminates GSCs and Induces Expression of Differentiation-Associated Proteins

To observe the effects of si-hVDAC1 silencing on GSCs, we followed several specific markers. These included nestin, a marker for CNS stem cells and SOX2, a member of SOX TF family that is predominantly expressed in immature and undifferentiated CNS cells and is important for neural stem cell proliferation and differentiation. KLF4, a member of the family of zinc-finger TFs that serve multiple functions; CD133, a cell-surface marker;[34,35] nerve growth factor receptor ([NGFR] p75NTR, CD271), which plays different roles in several pathways; and ALDH1, are also considered GSC markers.

U-87GM cells treated with si-hVDAC1 showed marked decreases in the expression of these GSC markers following the third and fourth transfections, as evaluated by immunoblot (Figures 3A and 3B) and real-time qPCR (Figure 3C), which also revealed decreases in nanog mRNA levels. Immunofluorescence (IF) staining of nestin in cells treated with si-hVDAC1 was decreased, relative to si-NT-treated cells (Figure 3D), as confirmed by immunoblot and real-time qPCR (Figure 3A, 3B and 3C).

The decrease in the levels of GSC markers upon metabolism reprograming may result from an arrest of cell proliferation (Figures 1G and 1H) and/or promotion of differentiation. Cell differentiation can be affected by altering cancer cell metabolism.[36] Indeed, VDAC1 silencing in U-87MG-derived tumors resulted in tumor cell differentiation into astrocyte- and neuron-like cells.[11] Here, we asked whether similar changes can be induced in U-87MG cells in culture when treated with si-hVDAC1.

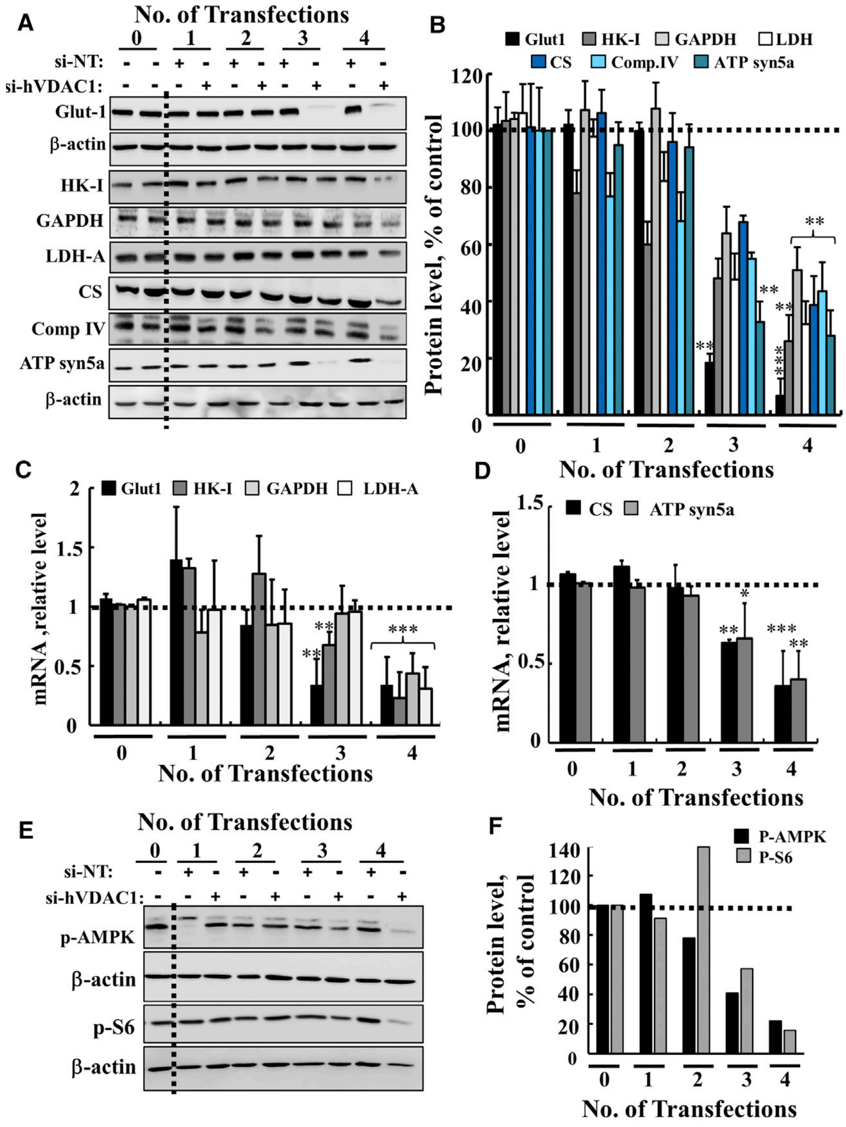

Figure 2. si-hVDAC1 Treatment of U-87MG Cells Resulted in Reprogramed Cell Metabolism after the Third and Fourth Transfections

(A and B) Immunoblot (A) and quantitative analysis (B) of selected metabolism-related proteins from si-NT- or si-hVDAC1-treated U-87MG cells at the indicted number of transfections, using specific antibodies against the metabolic enzymes Glut-1, HK-I, GAPDH, LDH-A, CS, complex IVc, and ATP 5a synthase. β-Actin served as an internal loading control. (C and D) Real-time qPCR analysis of mRNA levels of metabolic enzymes in si-hVDAC1-treated U-87MG cells, relative to those in si-NT-treated U-87MG cells, following the indicated number of transfections. Results reflect the mean ± SEM; *$p \leq 0.05$; **$p \leq 0.01$; ***$p \leq 0.001$. (E and F) Immunoblot (E) and quantitative analysis (F) of phosphorylated AMPK and S6 (p-AMP and p-S6, respectively) using antibodies specific to the phosphorylated form of each. Dashed lines indicate control levels.

Cells silenced for VDAC1 expression for 5–20 days were immunostained for the mature astrocyte marker glial fibrillary acidic protein (GFAP), as well as for the neuronal marker TUBB3 (Figures 4A–4D). Both proteins were found to be highly expressed in cells after 15 and 20 days of VDAC1 silencing (Figures 4A and 4B). Similar results were obtained at the mRNA level (Figure 4C). The expression of another neuronal marker, MAP2 was also increased after 15 and 20 days of VDAC1 silencing (Figure 4C). These findings were further supported by IF staining showing increased TUBB3 staining, whereas the stem cell marker, nestin staining decreased (Figure 4D), in correlation with immunoblot and real-time qPCR results (Figures 4A–4C).

Interestingly, cell morphological changes reflected in the appearance of processes were observed in the si-hVDAC1-treated cells (Figure 4D, enlargement). Next, cells subjected to a fourth transfection with si-NT- or si-hVDAC1 were stained with anti-ezrin antibodies (Figure 4E). Ezrin is a member of the ERM (ezrin-radixin-moesin) family of membrane-cytoskeleton-linking proteins, expressed in peripheral astrocytic processes in rat hippocampus, in primary cultured astrocytes[37] and in glial tube cells.[38] The results clearly showed changes in the morphology of si-hVDAC1-treated cells, showing long and marked processes that were not seen in the si-NT-treated cells (Figure 4E). Finally, podoplanin (PDPN) levels were decreased in si-VDAC1-treated cells, as revealed by IF (Figure 4F). PDPN expression has been described in various human tumors and has been linked to cytoskeleton regulation and thus to increased migration and invasion.[39] These results argue that si-hVDAC1 cell treatment over time led U-87MG cells to differentiate toward mature astrocyte- and neuron-like cells.

As the major TFs p53, hypoxia-inducing factor 1a (HIF-1α), and c-myelocytomatosis (c-Myc) are known to regulate metabolism, cell growth, proliferation, and differentiation,[40] we analyzed their expression levels in cells treated with si-hVDAC1 (Figure 5). Both immunoblot and real-time qPCR demonstrated that p53 expression levels were elevated in si-hVDAC1-treated cells, whereas those of HIF-1α and c-Myc were reduced as a function of the number of transfections (Figures 5A–5C). Although p53 levels were highly increased, no apoptotic cell death was observed (data not shown).

Levels of the pro-apoptotic protein cytochrome *c* (Cyto *c*) were highly increased in the si-hVDAC1-treated cells as a function of the length of time that the cells were depleted of VDAC1, as revealed

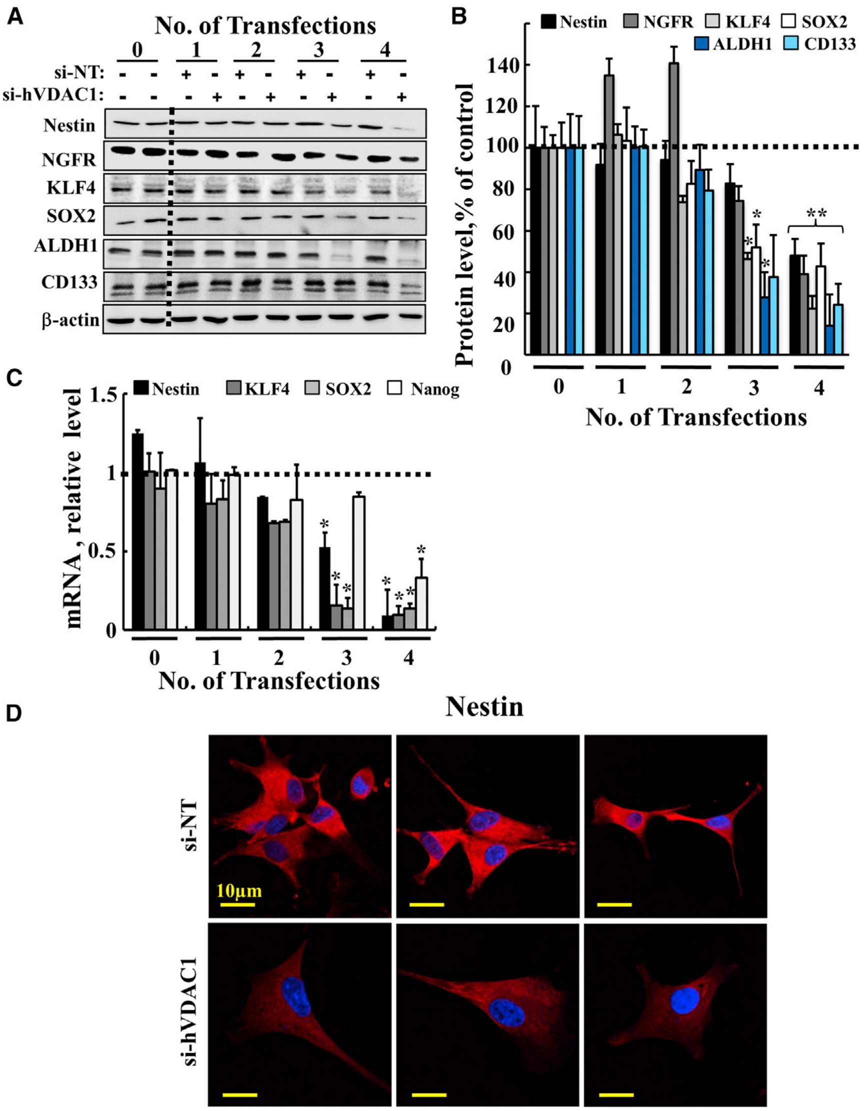

Figure 3. si-hVDAC1 Treatment Markedly Reduced Cancer Stem Cell Marker Expression in the U-87MG Cell Line after the Third and Fourth Transfections

(A) Immunoblots of selected neuronal stem cell markers (nestin, NGFR, KLF4, SOX2, ALDH1, the CD133) in protein extracts of U-87MG cells treated with si-NT or si-hVDAC1 for the indicated number of transfections using specific antibodies. β-Actin served as an internal loading control. (B) Quantitative analysis of immunoblots of 4 experiments as in (A) is shown. Results reflect the mean ± SEM; $*p \leq 0.05$; $**p \leq 0.01$. (C) Real-time qPCR of mRNA levels of CSC markers (nestin, KLF4, SOX2, and nanog) in U-87MG cells treated with si-NT or si-hVDAC1 for the indicated number of transfections, and presented relative to their levels in si-NT-treated cells. Results reflect the mean ± SEM; $*p \leq 0.05$. The dashed line indicates the level in the controls. (D) Immunofluorescent staining for nestin in si-NT- and si-hVDAC1-treated cells after the fourth transfection examined. Cells were visualized by confocal microscopy (Olympus IX81).

by immunoblot and real-time qPCR (Figures 5D and 5E). This finding can be explained by the association of Cyto *c* with cell differentiation and remodeling of nuclear chromatin.[41,42]

The results presented in Figures 1, 2, 3, 4, and 5 are summarized in Table 1, presenting the complex set of effects of VDAC1 depletion on a network of key regulators of cell metabolism, leading cancer cells toward differentiation. Most important, a reciprocal relationship between the decrease in stem cell markers and an increase in differentiation markers was obtained (Table 1).

VDAC1 Silencing Leads to Rewired Metabolism in the A549 Lung Cancer and MBA-MD-231 Triple-Negative Breast Cancer Cell Lines

Next, we asked whether similar effects of si-hVDAC1 as seen with U-87MG cells would also be obtained with other cancer cell lines, such as the A549 lung cancer and MBA-MD-231 TNBC cell lines (Figures 6, 7, and 8; Tables S1 and S2). A549 is a non-small cell lung carcinoma cell line derived from a primary tumor, and the cells are characterized as pre-alveolar type II pneumocytes of human lung and carry several mutations (i.e., RAS, CDKN2A, FLT3, CBL, KEAP1, ZFHX3, FH, FUS, STK11, ATR, SUFU, HIP1, and SMARCA4). MDA-MB-231 cells are derived from a pleural effusion metastatic tumor and correspond to a poorly differentiated TNBC cell line that does not express the ER, PR or ERBB2/Her2.[15] These cells carry several mutations (i.e., BRAF, RAS, CDKN2A, TP53, PTEN, BRIP1 and LIFR). For comparison, the U-87MG cell line represents glioblastoma hypodiploid cells derived from a primary brain tumor and carries several mutations (i.e., CDKN2A, RAS, PTEN, HF1 and PCM1).

As with U-87MG cells, si-hVDAC1 treatment of A549 or MDA-MB-231 cells was carried out four times every 5 days (Figure 6A), with VDAC1 expression being decreased from the first transfection (Figures 6B and 6C). si-RNA downregulated VDAC1 but not VDAC2 or -3 levels (Figures 6D and 6E). Mitochondria $\Delta\psi$ (Figure 6F) and cellular ATP levels (Figure 6G) were also decreased in both si-hVDAC1-treated cell lines.

Immunoblot and real-time qPCR analyses indicated that the energy-production-related enzymes Glut-1, HK-I, GAPDH, LDH-A, CS, electron transport subunit IVc, and ATP synthase 5a were reduced following the third and fourth transfections of MDA-MB-231 (Figures 7A–7C) and A549 (Figures S1A and S1B) cells.

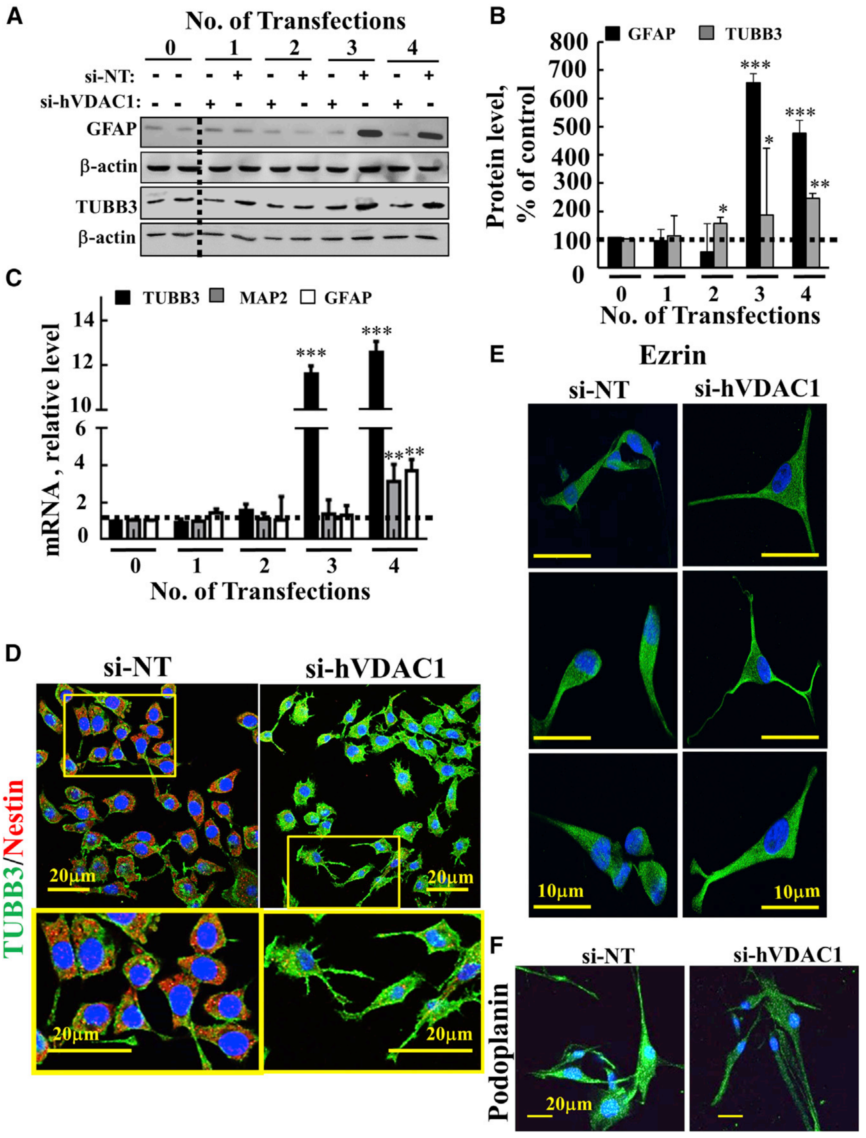

Figure 4. si-hVDAC1 Treatment of U-87MG Cells Induced Expression of Proteins Associated with Differentiation Following the Third and Fourth Transfections

(A and B) Immunoblot (A) and quantitative (B) analyses of selected differentiation markers using specific antibodies (anti-GFAP and anti-TUBB3) of si-NT- or si-hVDAC1-treated U87-MG cells. β-Actin served as an internal loading control. (C) Real-time qPCR analysis of mRNA levels in si-hVDAC1-treated cells, relative to those in si-NT-treated cells, of selected differentiation markers using specific primers for TUBB3 (black bar), MAP2 (gray bar), and GFAP (white bar). Results reflect the mean ± SEM; $^{*}p \leq 0.05$; $^{**}p \leq 0.01$; $^{***}p \leq 0.001$. Dashed lines indicate the controls level. (D) Immunofluorescence staining of nestin and TUBB3 in si-NT- and si-hVDAC1-treated cells after the fourth transfection. (E and F) IF staining of ezrin (E) and of podoplanin (F) in si-NT- and si-VDAC1-treated U-87MG cells after the fourth transfection. Cells were visualized by confocal microscopy (Olympus IX81).

Also, as with U-87MG cells, expression levels of p53 were highly elevated, whereas those of HIF-1α and c-Myc were reduced in the si-hVDAC1-treated MDA-MB-231 (Figures 7D and 7E) and A549 (Figures S1C and S1D) cells, as a function of the number of transfections.

Cell Treatment with si-hVDAC1 Eliminates CSCs and Induces Expression of Differentiation-Associated Proteins in MDA-MB-231 Breast and A549 Lung Cancer Cells

The effects of VDAC1 silencing in MDA-MB-231 cells on CSCs and differentiation was analyzed by following several specific markers of CSCs and differentiation.[43] The expression of the CSC markers CD133, ALDH1, SOX2, and KLF4 were markedly decreased after the third and fourth transfections (Figures 8A and 8B). $CD44^{+}$/$CD24^{-}$ breast cancer cells have stem/progenitor cell properties.[44] In cancerous cells, CD44, a receptor of hyaluronan, metalloproteinases, and collagen types I and IV, is highly expressed, whereas CD24 is absent or expressed at low levels. si-hVDAC1 treatment of MDA-MB-231 cells resulted in downregulation of CD44 levels and upregulation of CD24 expression levels (Figure 8C).

MDA-MB-231 cells IF stained for HER2 showed increased expression levels in si-hVDAC1-treated cells, relative to its expression levels in si-NT-treated cells (Figure 8D). The expression levels of prolactin (PRL), ER, and PR were all increased in si-hVDAC1-treated cells (Figure 8E). Interestingly, PRL mRNA levels were highly increased (6- to 8-fold) following the first transfection with si-hVDAC1 (Figure 8E).

A549 cells showed decreased expression of CD133, ALD1H, SOX2, KLF4, CD44, and ABCG2, following the third and the fourth treatments with si-hVDAC1, as evaluated by immunoblot analysis (Figures S2A and S2B). IF staining confirmed the decreased expression of CD44 and ABGG2 (Figure S2C).

A549 cells are considered to be not fully differentiated alveolar epithelial type II (AT2) cells.[45] AT2 cells are surfactant-producing cells expressing the pulmonary-associated surfactant proteins (SFTP) A, B, C, and D. IF staining of SFTP C expression was increased in cells treated with si-VDAC1 (Figure S2D).

AT2 cells can be differentiated into pulmonary alveolar type I (AT1) cells, characterized by the expression of podoplanin (PDPN), a membranal mucin-type sialoglycoprotein. PDPN expression levels were reduced after the first si-hVDAC1 transfection and remained low after subsequent transfections (Figure S2D and S2E). At the same time,

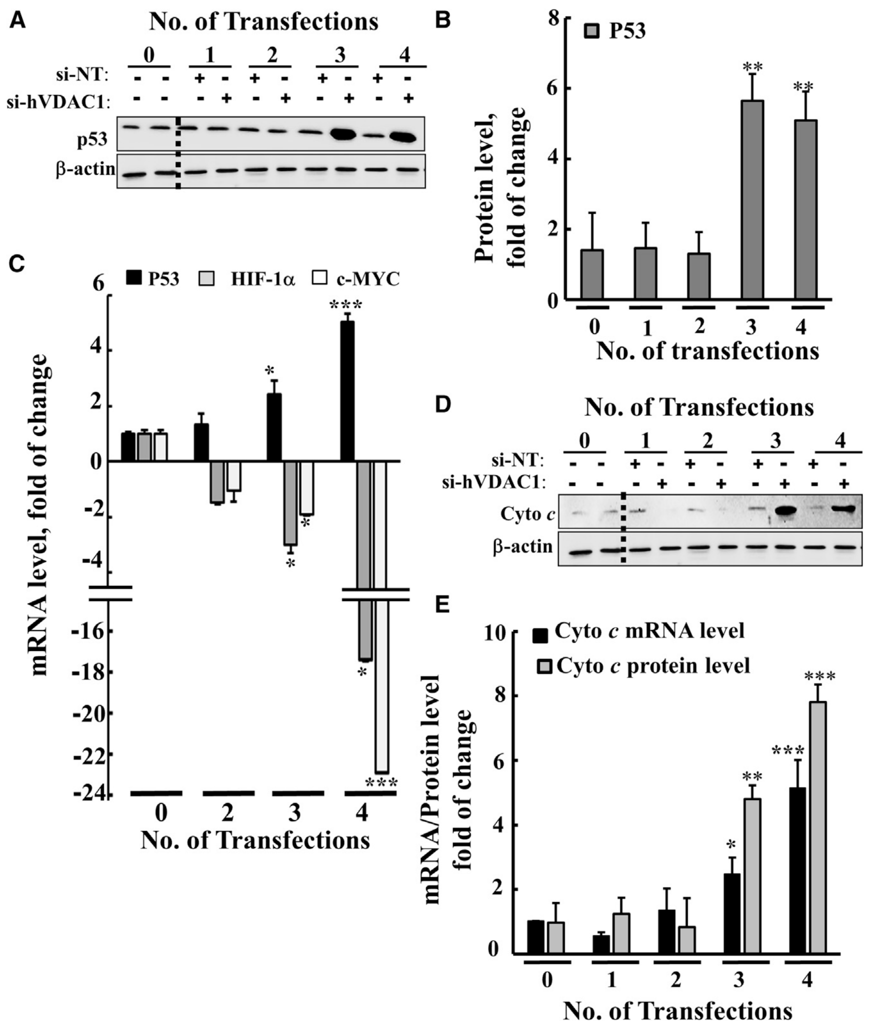

Figure 5. si-hVDAC1 Treatment Markedly Altered Transcription Factor Expression in U-87MG Cells after the Third and Fourth Transfections

(A and B) Immunoblot (A) and quantitative (B) analyses of p53 levels in protein extracts from si-NT- or si-hVDAC1-treated U-87MG cells after the indicated number of transfections. (C) Real-time qPCR analysis of mRNA levels, at the indicated transfection, of p53, c-Myc, and HIF-1α, using specific primers. The fold change in mRNA levels in si-hVDAC1-treated U-87MG cells, relative to those in si-NT-treated U-87MG cells, is presented. (D and E) Immunoblot of Cyto *c* in si-hVDAC1-treated U-87MG cells at the indicated transfection (D). Real-time qPCR analysis of Cyto *c* levels in si-hVDAC1-treated U-87MG cells, relative to those in si-NT-treated U-87MG cells (E). Results reflect the mean ± SEM, *$p \leq 0.05$; **$p \leq 0.01$; ***$p \leq 0.001$.

the levels of another AT1 marker, homeodomain only protein x (HOPX), were reduced only after the fourth transfection (Figure S2E). These results suggest that the non-fully differentiated A549 cells had undergone reprograming, as reflected in alterations in the expression of several proteins characterizing AT1 or AT2 cells.

The results obtained upon reprograming of cell metabolism, leading cancer cells toward differentiation, obtained with A549 and MDA-MB-231 cells, are summarized in Tables S1 and S2, showing, as with GBM, a reciprocal relationship between the decrease in CSCs and cell differentiation.

DISCUSSION

In this study, we demonstrated that metabolic reprograming of GBM and lung and breast cancer cells resulted in a sequence of events leading to cell differentiation into a less malignant state. We kept VDAC1 expression at low levels over time by repeated cell transfection with si-hVDAC1. The obtained results demonstrated that the cell reprograming process induced by VDAC1 depletion, as reflected in alterations in the expression of proteins and mRNA, is dynamic over time. This time dependency was observed for both downregulated proteins, such as metabolic enzymes, CSC markers, and some TFs, and upregulated proteins, such as p53 and some of the proteins associated with cell differentiation. This reciprocal relationship suggests a link between the decrease in energy, cell growth and stem cell levels, and induced differentiation, as also found in the tumor mouse model,[11,46] with its microenvironment.

Reprograming of Cancer Cell Metabolism Induced by Silencing VDAC1 Expression

The Warburg phenotype was originally associated with weak mitochondrial activity. However, mitochondrial metabolism plays a crucial role in cancer cell survival and development, including the use of substrates, such as glutamine and/or fatty acids,[47] for ATP biosynthesis, and encompasses macromolecular biosynthesis. Given the role of mitochondria in cancer cell fate, the potential to interfere with mitochondrial function is a promising avenue for treating cancer.

Here, we demonstrated such a strategy using downregulation of VDAC1 expression in cancer cells. As metabolism represents an assimilation of several signals from multiple coordinated pathways, with the mitochondria at the heart and VDAC1 being the mitochondrial gatekeeper, it is not surprising that downregulation of VDAC1 resulted in reprogramed cell energy homeostasis, leading to reduced cell function and induced differentiation. We showed that depleting VDAC1, which is overexpressed in many tumors, including GBM and lung and breast cancers,[7,9,46] resulted in a decreased expression of glycolysis-, Kreb's cycle-, and OXPHOS-related enzymes, reflecting reduced anaplerotic and OXPHOS reactions.

Table 1. Summary of the Expression Levels of Genes Associated with Metabolism, Stem Cells, Differentiation, and Transcription Factors as a Function of the Number of Transfections of U-87MG Cells with si-VDAC1, Relative to Transfection with si-NT (qPCR Results)

Gene	Fold of Change si-NT/si-VDAC1			
Transfection No.	0	2	3	4
Metabolism				
Glut-1	1.00 ± 0.03	−1.2 ± 0.13	−2.90 ± 0.21	−3.1 ± 0.23
HK-I	1.00 ± 0.01	−1.27 ± 0.31	−1.5 ± 0.11	−4.4 ± 0.21
LDH-A	1.00 ± 0.01	−1.2 ± 0.28	−1.4 ± 0.08	−3. 2 ± 0.17
CS	1.00 ± 0.01	1.0 ± 0.14	−1.6 ± 0.02	−2.8 ± 0.22
ATP synthase 5a	1.00 ± 0.01	−1.10 ± 0.06	−1.6 ± 0.22	−2.5 ± 0.17
VDAC1	1.00 ± 0.03	−2.3 ± 0.2	−2.8 ± 0.23	−3.3 ± 0.07
VDAC2	1.00 ± 0.13	1.2 ± 0.08	1.3 ± 0.32	1.1 ± 0.04
VDAC3	1.00 ± 0.08	1.11 ± 0.07	0.90 ± 0.11	1.2 ± 0.04
Stem Cell				
Nestin	1 ± 0.12	−1.17 ± 0.07	−1.9 ± 0.3	−15.6 ± 0.05
KLF4	1 ± 0.08	−1.5 ± 0.03	−3.9 ± 0.04	−7.5 ± 0.06
SOX2	1 ± 0.09	−1.2 ± 0.03	−5.2 ± 0.06	−5.1 ± 0.03
Nanog	1 ± 0.01	−1.2 ± 0.22	−1.2 ± 0.02	−3 ± 0.21
Differentiation				
TUBB3	1 ± 0.03	1.6 ± 0.32	11.2 ± 0.7	13.2 ± 0.96
MAP2	1 ± 0.02	1 ± 0.08	2.5 ± 0.37	3.2 ± 0.18
GFAP	1 ± 0.01	1 ± 1.25	1.9 ± 0.55	3.7 ± 0.61
Cyto *c*	1 ± 0.06	1.4 ± 0.38	2.5 ± 0.48	5.1 ± 0.28
TFs				
p53	0.9 ± 0.06	0.9 ± 0.08	1.20 ± 0.08	4.70 ± 0.10
c-Myc	1.0 ± 0.12	1.07 ± 0.04	1.02 ± 0.05	−21. 4 ± 0.01
HIF1-α	1.25 ± 0.12	−1.17 ± 0.07	−2.40 ± 0.30	−13.9 ± 0.08

The changes in cell metabolism and energy production, seen upon suppressing VDAC1 expression, were also reflected in the decrease in activated AMPK levels, whose activity increases during metabolic stress conditions, controlled by ATP restriction.[30] In this respect, AMPK has been demonstrated to be highly activated *in vivo* in human and rodent glioblastoma.[48] Moreover, activation of AMPK can be a pro-tumorigenic signal in cancer and hence a possible therapeutic target in cancer treatment.[29] Furthermore, AMPK regulates many TFs, their co-activators, and histones to stabilize gene expression and nuclear events, which leads to cell survival and metabolic reprograming.[30] Finally, AMPK is required to support tumor growth in murine Kras-dependent lung cancer models.[49] Thus, these reports agree with our results showing that in the three cancers considered, high levels of activated p-AMPK in si-NT-treated cells were reduced significantly upon transfection with si-hVDAC1. Given that VDAC1 depletion rewires cancer cell metabolism and reverses cancer cell properties to normal-like cells, the decrease in activated AMPK levels is expected.

VDAC1 depletion also affected the mTOR pathway that senses a cell's energetic status and nutrient and oxygen levels to regulate cell growth and survival,[33,50] as reflected in decreased levels of phosphorylated S6. Thus, metabolic reprograming involves downregulation of metabolism-related enzymes and affects metabolic control via the mTOR pathway and AMP-activated protein kinase. Moreover, VDAC1 deletion resulted in similar reprograming of cell metabolism in the three types of cancer cell lines addressed here (GBM and lung and breast cancers), regardless of cellular origin or mutations carried. Furthermore, similar results were obtained as with sub-cutaneous U-87MG, A549, and MDA-MB-231 cell-derived xenograft mouse models,[46] suggesting that the observed reprograming is not tumor microenvironment dependent, but instead involves an intrinsic cell machinery. Finally, the reprograming of cancer cells is a process that develops over time; although VDAC1 expression was highly reduced one day after si-hVDAC1 treatment, the altered expression of proteins was seen after 15–20 days of the cells being depleted of VDAC1.

Reprogramed Metabolism Eliminates CSCs, Possibly Via Promoting Their Differentiation

CSCs are undifferentiated cancer cells with self-renewal capacity and thus possess high tumorigenic activity and are associated with tumor resistance to anti-cancer treatments, the major cause of cancer recurrence and metastasis.[51] Thus, eliminating CSCs or induction of their differentiation is required for complete eradication of tumors. Several therapeutic approaches that target CSCs via disrupting their quiescence or their resistance to oxidative stress have been proposed.[51]

Here, we presented a novel mechanism for targeting CSCs that results from the rewiring of cancer cell metabolism, leading to elimination of CSCs, simultaneously leading to differentiation. The negative correlation between CSC disappearance and the appearance of differentiated cells, observed in cells depleted of VDAC1 for 15–20 days, suggests that the differentiated cells originated from CSCs.

What leads to CSC differentiation? CSCs possess a multilineage differentiation potential and can undergo dynamic and reversible changes, depending on the surrounding microenvironment, in a process defined as dynamic stemness.[52] CSCs can undergo hyper-adaptation to the tumor microenvironment, as seen under hypoxia.[53] Here, however, we showed that differentiation occurred not only in tumors,[11,46] but also in cells in culture, suggesting that differentiation of CSCs is an intrinsic property that does not absolutely require or is induced by the microenvironment.

We demonstrated that, as in *in vivo* U-87MG-derived GBM tumors,[11,46] the same cells originating from astroglia in culture undergo differentiation into astrocyte- and neuron-like cells. We also showed that U-87MG cell differentiation developed with time, as even when VDAC1 was downregulated following the first and second transfections with si-VDAC1, differentiation neuronal markers, such as GFAP and TUBB3, appeared only after

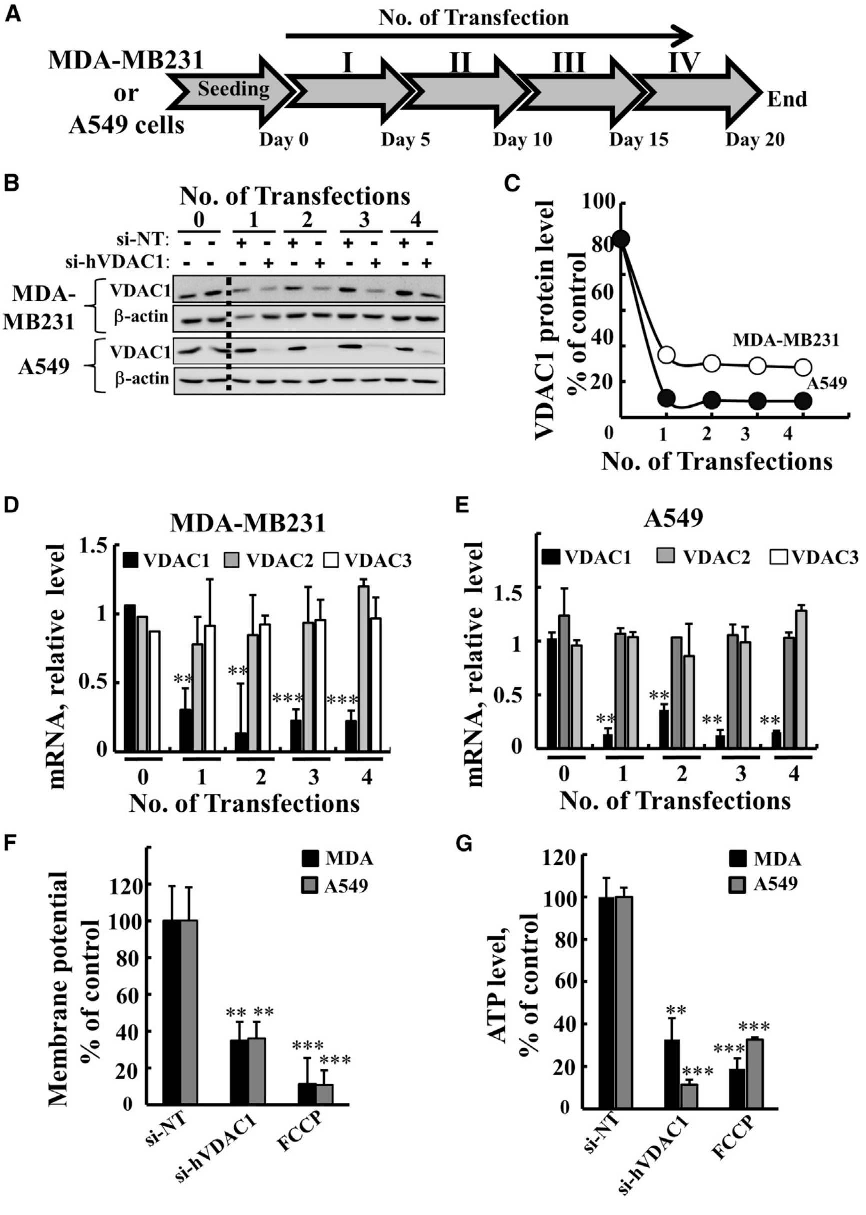

Figure 6. si-hVDAC1 Treatment of the MDA-MB-231 and A549 Cell Lines Silenced VDAC1 Expression, Inhibited Cell Growth and Reduced Energy Production

(A) Protocol for si-NT and si-hVDAC1 treatment of the MDA-MB-231 and A549 cell lines. Cells (150,000 cells/well in 6-well plates) were transfected with either 50 nM of si-NT or si-hVDAC1 four times every 5 days. (B and C) VDAC1 expression levels in MDA-MB-231 and A549 cells following the indicated transfection were analyzed by immunoblot (B) and quantification (C). (D and E) mRNA expression levels of VDAC1, -2, and -3 in the MDA-MB-231 (D) and A549 (E) cell lines following the indicated number of transfections, as analyzed using real-time qPCR and specific primers. (F and G) MDA-MB-231 (black bar) and A549 (gray bar) cells were analyzed for mitochondrial membrane potential (ΔΨ) (F) and ATP (G) levels following the third transfection. Results reflect the mean ± SEM; $^{**}p \leq 0.01$; $^{***}p \leq 0.001$.

the third and fourth transfections with si-hVDAC1 (15–20 days of low VDAC1 levels), concomitant with CSC elimination. Differentiated astrocyte- or neuron-like cells in culture were also reflected by the cells exhibiting neuronal morphology, as well as decreased expression of factors associated with cancer, such as PDNP.

Reprograming human glioma cells so as to convert them into terminally differentiated neuron-like cells in both culture and in adult mouse brain was demonstrated via expression of a TF functioning in neurogenesis, neurogenin 2 (NGN2), in synergy with SOX11.[54] Our results thus suggest that glioma cells possess differentiation potential that can be activated by reprograming metabolism affecting TFs and the gene expression program.

Mammary CSCs are defined by a $CD44^{+-}CD24^{-}/^{low}$ phenotype. The expression of CD44, positively associated with stem cell-like characteristics was decreased, whereas that of CD24, the expression of which is related to differentiated epithelial features,[55] was increased in si-hVDAC1-treated MDA-MB-231 cells. Furthermore, the $CD44^{+}CD24^{-}/^{low}$ and $ALDH1^{+}$ phenotypes were proposed to identify CSCs with distinct levels of differentiation.[56]

In the TNBC (ER^{-}, PR^{-}, and $HeER2^{-}$) MDA-MB-231 cells, si-hVDAC1 treatment increased the expression levels of prolactin, ERs, PRs, and HER2. These changes suggest differentiation into less malignant lineages. In this respect, although existing treatment modalities are effective in treating early breast cancer, they have limited usefulness in treating the TNBCs (ER^{-}, PR^{-}, and $HER2^{-}$). The induction of expression of these receptors in the TNBC cells upon VDAC1 depletion opens the way for hormonal and HER2-based therapy for such patients.

Similarly, we showed that, upon VDAC1 depletion in the A549 lung cancer cell line, representing non-mature AT2 cells, the expression of SFTP C was increased after the third and fourth transfections. In the case where AT2 cells differentiated into AT1 cells, we expected increased expression of PDPN, a mucin-type glycoprotein. On the contrary, PDPN expression was decreased in VDAC1-depleted cells, in line with PDPN's presence in many types of normal cells, such as endothelial cells in lymphatic vessels, and not only in AT1 cells.[57] In numerous types of human carcinomas, PDPN is often upregulated, particularly in squamous cell carcinomas, such as cervical, skin, and lung cancers.[57,58]

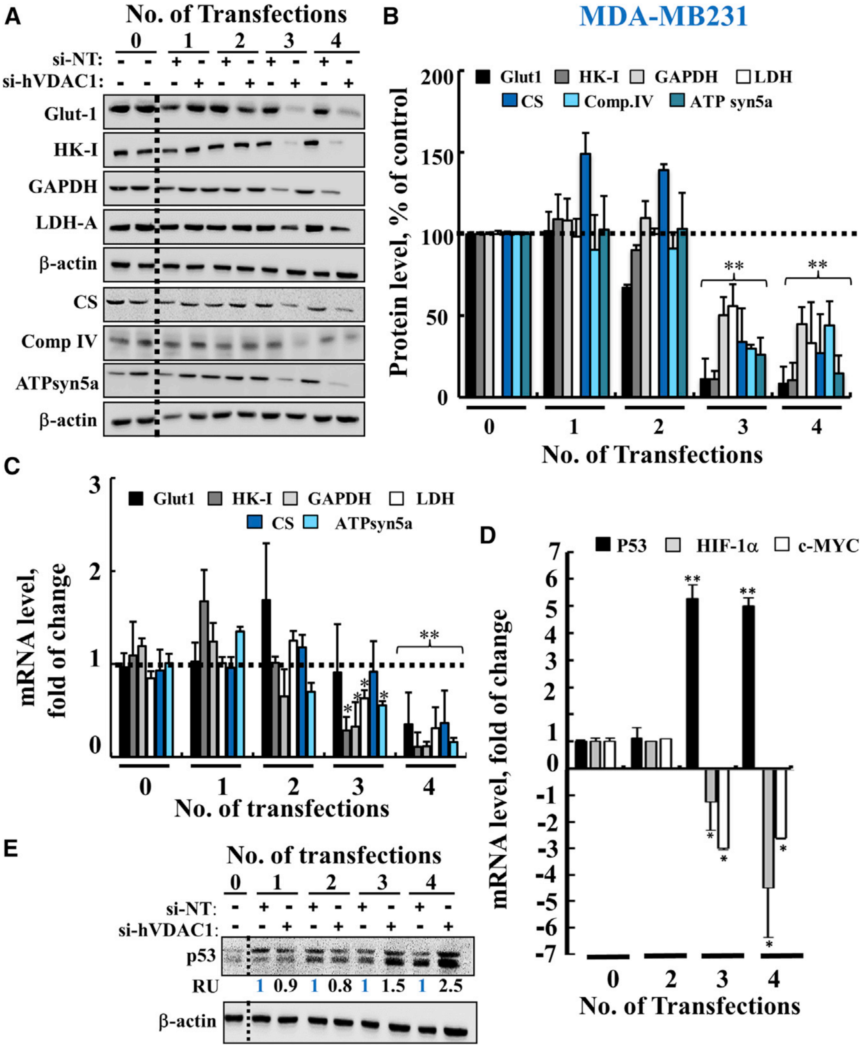

Figure 7. si-hVDAC1 Treatment of MDA-MB-231 Cells Reprogramed Metabolism and Altered TF Expression after the Third and Fourth Transfections

(A and B) Immunoblot (A) and quantitative analyses (B) of Glut-1, HK-I, GAPDH, LDH-A, CS, complex IVc and ATP 5a synthase in si-NT- or si-hVDAC1-treated MDA-MB-231 cells following the indicated transfection. β-Actin served as an internal loading control. (C and D) Real-time qPCR analysis of mRNA levels of metabolic enzymes (C) and the TFs p53 (black bar), HIF-1α (gray bar), and c-Myc (white bar) (D) in si-hVDAC1-treated MDA-MB-231 cells, relative to those in si-NT-treated MDA-MB-231 cells, following the indicated transfection. Results reflect the mean ± SEM; $^*p \leq 0.05$; $^{**}p \leq 0.01$. The dashed line indicates the level in the controls. (E) Immunoblot analysis of p53 levels in si-NT- and si-hVDAC1-treated MDA-MB-231 cells following the indicted transfection. Quantitative analysis of the immunoblots is presented as average relative units (RU) of the indicated protein in si-hVDAC1-treated relative to si-NT-treated MDA-MB-231 cells. β-Actin served as an internal loading control.

PDPN is believed to play a key role in cancer cell invasiveness by controlling invadopodia, thus mediating efficient extracellular matrix degradation.[59] It has been associated with poor prognosis[60] and is also found on the surface of cancer-associated fibroblasts (CAFs) in lung adenocarcinomas, as well as in breast and pancreatic tumors, brain tumors, and other cancers.[61] CAFs are correlated with an increased incidence of metastasis to lymph nodes and shorter survival times of patients.[57] PDPN is considered as a specific lymphatic vessel marker, and since lymphangiogenesis levels are correlated with poor prognosis in cancer patients, it is proposed as a diagnostic marker.[58] Finally, PDPN is expressed in invasive neuronal cancer stem cells.[58] Our findings that PDPN expression is reduced in si-hVDAC1-treated cells is in line with the above pro-cancer functions of PDPN. si-hVDAC1 thus acts as an antagonist to such PDPN pro-cancer activity.

The complex set of effects resulting from VDAC1 depletion on a network of key regulators of cell metabolism, CSCs, TFs, and other factors, eventually leading cancer cells toward differentiation, indicates that all these changes are not random but rather occur as an ensemble. The changes, reflected as either up- or downregulation, occurred at the same time, starting at 15 days and further developing after 20 days, during which time VDAC1 levels were maintained at low levels (Tables S1 and S2).

Reprogramed Metabolism Altered TF Expression Levels and Signaling Pathways

During tumorigenesis, de-regulated TF expression or activation can promote CSC self-renewal, proliferation, and differentiation. Differentiation is a regulated process controlled via gene regulatory networks that involve TFs and epigenetics. Cell differentiation driven by reprograming of metabolism as brought about by VDAC1 depletion is mediated via TFs, and pathways are regulated at the energy level. p53, HIF-1α, and c-Myc were shown to regulate cell metabolism, growth, proliferation, and differentiation.[40]

Starting at 15 days and further developed after 20 days of low VDAC1 levels, p53 levels were increased, whereas HIF-1α and c-Myc expression levels were reduced. p53 contributes to a diverse range of cellular functions, including regulation of DNA repair, cell cycle progression, and regulation of senescence, apoptosis differentiation, and cellular metabolism, by directly regulating glucose metabolism and OXPHOS.[62,63] p53 is implicated in the regulation of differentiation in skeletal muscle, neurons, hematopoietic cells, and adipocytes.[64] Thus, the increase in p53 expression levels seen in the three cancer cell lines tested after the third and fourth transfections with si-hVDAC1 is in accord with the function of p53 in regulating many functions, such as cellular metabolism and cell differentiation.

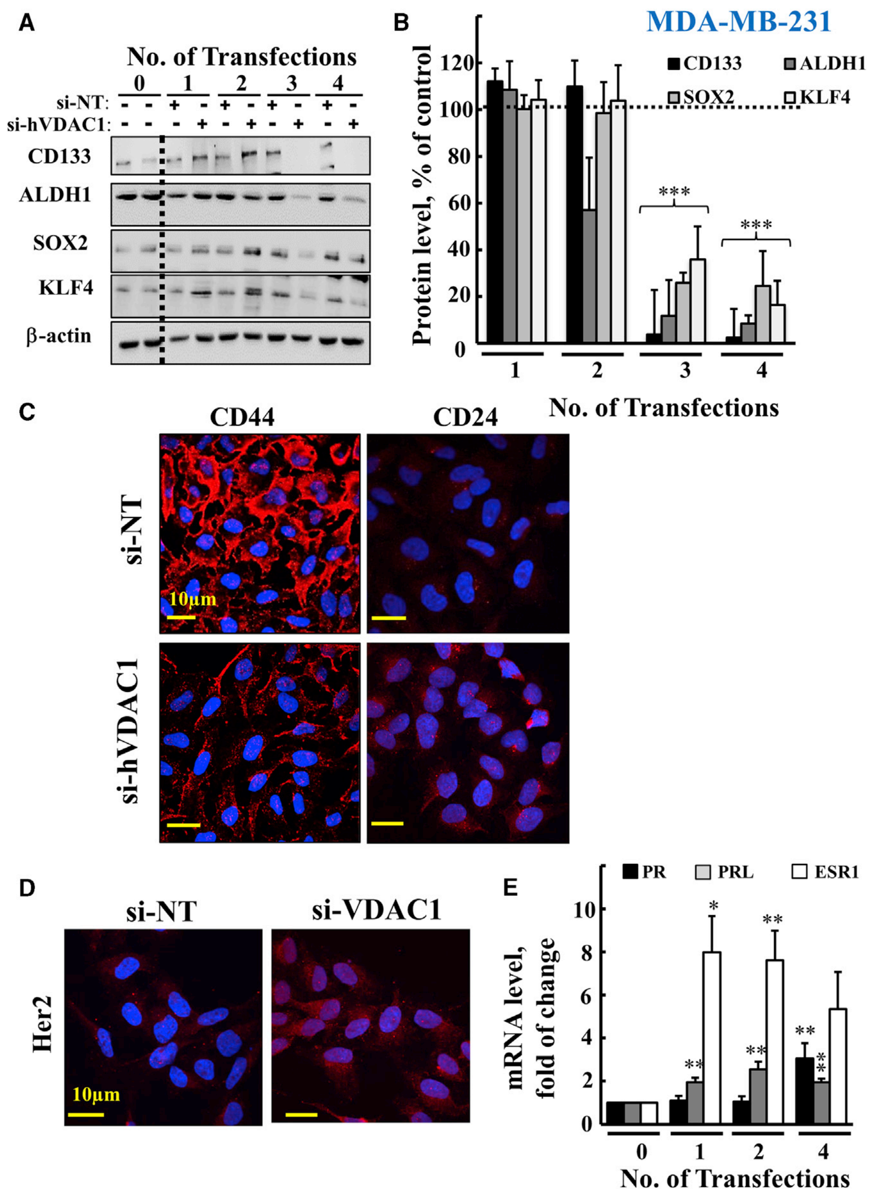

Figure 8. si-hVDAC1 Treatment of MDA-MB-231 Cells Markedly Reduced Expression of CSC Markers and Enhanced Expression of Proteins Associated with Differentiation after the Third and Fourth Transfections

(A and B) Immunoblot (A) and quantitative analyses (B) of selected CSC markers (CD133, ALDH1, SOX2, and KLF4) in si-NT- and si-hVDAC1-treated MDA-MB-231 cells following the indicated transfection, using specific antibodies. β-Actin served as an internal loading control. The dashed line indicates the level in the controls. (C and D) IF staining of si-NT- and si-hVDAC1-treated cells following the fourth transfection stained for CD44 and CD24 (C) and, after the third transfection for Her2 (D), as visualized by confocal microscopy (Olympus IX81). (E) Real-time qPCR analysis of mRNA levels for progesterone receptor (PR; black bar), prolactin (PRL, gray bar), and estrogen receptor (ESR1, white bar) in si-hVDAC1-treated MDA-MB-231 cells, relative to those in si-NT-treated cells, following the indicated transfection. Results reflect the mean ± SEM; $*p \leq 0.05$; $**p \leq 0.01$; $***p \leq 0.001$.

HIF-1α is mainly subjected to post-translational regulation via proteasome-mediated degradation.[65] The increased stability and activation of HIF-1α in cancer cells contributes to altered glycolytic metabolism, invasion, and metastasis.[66] The decrease in HIF-1α in si-hVDAC1-treated cell lines is thus in agreement with the observed inhibition of pro-tumorigenic properties by such treatment.

c-Myc is a TF that targets a large network of genes involved in cellular processes, such as the regulation of cell growth, proliferation, differentiation, and metabolism.[40,67] c-Myc-mediated actions include driving the cellular program for glycolysis and glutaminolysis and allowing glucose-derived citrate to be used in lipid synthesis.[68] By binding to E-boxes and recruiting histone acetyltransferases, c-Myc is capable of controlling the expression of more than 15% of human genes. The decreased c-Myc levels in cells with reduced VDAC1 levels is in line with the reprogramed metabolism of these cells.

Thus, the altered expression of p53, c-Myc, and HIF1α resulting from VDAC1 silencing represents a central axis of cell metabolism, proliferation, and differentiation control.

In summary, the results presented here and summarized in Figure 9 show that, by downregulating VDAC1 in GBM and lung and breast cancer, we re-programmed cell energy, metabolism, and other cell functions essential for cancer cell survival. Moreover, the effects of VDAC1 depletion on a network of key regulators of cell metabolism, cancer stem cells, TFs, and other factors, finally leading to differentiation, are coordinated and are common to GBM and lung and breast cancer cell lines, despite differing in origin and carried mutations. These alterations are interconnected, as reflected in the findings that changes in protein expression levels, either the up- or downregulated, occurred simultaneously, starting after 15–20 days, during which time VDAC1 levels were maintained at low levels. Furthermore, we demonstrated that VDAC1 represents an intersection between metabolism and cancer biology, with metabolism reprograming after VDAC1 silencing, not only inhibiting cell proliferation, but also directing the cell toward a differentiated state. Thus, our study showed that VDAC1 depletion represents a trigger for reprograming malignant cancer cells into a post-mitotic state and probably into terminally differentiated cells and that this might be a promising therapeutic approach for various cancers.

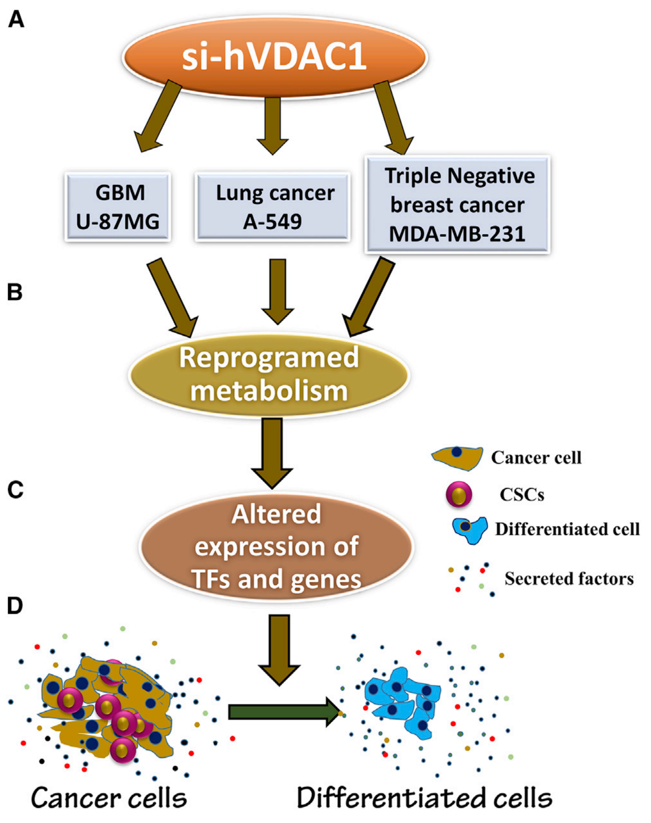

Figure 9. A Schematic Presentation of VDAC1 Depletion in GBM and Lung and Breast Cancer Cells, Leading to Metabolic Reprograming, Altered Expression of TFs and Genes, and Cell Differentiation

Silencing VDAC1 expression in the three tested cancer cell lines, regardless of their origin or carried mutations (A), resulted in reprograming of metabolism (B). This led to altered expression of master metabolism regulators (p53, HIF1-α, and c-Myc) and various genes (C) and to elimination of CSCs, while leading to cell differentiation (D).

MATERIALS AND METHODS

Materials

The cell transfection agents JetPRIME and JetPEI were obtained from PolyPlus-transfection (Illkirch, France), 2′-O-methyl-modified NT and hVDAC1-targeting siRNA were obtained from Genepharma (Suzhou, China). BSA, carbonyl cyanide-p-trifluoromethoxyphenylhydrazone (FCCP), SRB, Triton X-100, Tween-20, tetramethylrhodamine methylester (TMRM), and hematoxylin and eosin were obtained from Sigma (St. Louis, MO). Paraformaldehyde was purchased from EMSDIASUM (Hatfield, PA). DMEM was obtained from GIBCO (Grand Island, NY). Normal goat serum (NGS), fetal calf serum (FCS), and the supplements L-glutamine and penicillin-streptomycin were obtained from Biological Industries (Beit Haemek, Israel). Primary antibodies, their sources, and the dilutions used are detailed in Table S3. Horseradish-peroxidase (HRP)-conjugated anti-mouse, anti-rabbit, and anti-goat antibodies were from KPL (Gaithersburg, MD). 3,3-Diaminobenzidine (DAB) was obtained from ImmPact-DAB (Burlingame, CA).

Cell Culture and Transfection

U-87MG (human glioblastoma), MDA-MB-231 (human breast carcinoma), and A549 (non-small lung carcinoma) cells were maintained at 37°C and 5% CO_2 in the recommended culture medium and supplements. 2′-O-methyl-modified si-NT and si-hVDAC1/2A were synthesized by Dharmacon or were obtained from Genepharma. The sequences used with 2′-O-methyl-modified nucleotides underlined and sense (S) and anti-sense (AS) sequences are:

si-NT, S: -5′-GCAAACAUCCCAGAGGUAU-3′ and AS: 5′-AUACCUCUGGGAUGUUUGC-3′; si-hVDAC1 2/A, S: 238-5′-ACACUAGGCACCGAGAUUA-3′-256 and AS: 238-5′-UAAUCUCGGUGCCUAGUGU-3′. Cells were seeded (150,000 cells/well) in 6-well culture dishes to 40%–60% confluence and the first transfection with 50 nM of si-NT or si-hVDAC1/2A was carried using the JetPRIME transfection reagent, according to the manufacturer's instructions. The second, third, and fourth transfections were carried out every 5 days.

SRB Assay for Cell Proliferation

U-87MG, A549, and MDA-MB-231 cells transfected with si-NT or si-hVDAC1 one, two, three, or four times were counted and seeded in 96-well plates. After an additional 48 h, the cells were stained with SRB, as described previously.[11]

Immunoblot and IF

For immunostaining, proteins in cell lysates resolved by SDS-PAGE and electro-transferred to nitrocellulose membranes. These were blocked with 5% non-fat dry milk and 0.1% Tween-20 in Tris-buffered saline (TBST), and incubated with primary followed by secondary antibodies (sources and dilutions as detailed in Table S3). Enhanced chemiluminescence was used for detection of horseradish peroxidase activity. Band intensity quantitation was performed using FUSION-FX (Vilber Lourmat, Marne-la-Vallée, France) software, and values were normalized to the intensities of the appropriate β-actin signal that served as a loading control.

U-87MG, A549, and MDA-MB-231 cells, following transfection with si-NT or si-hVDAC1 one, two, three, or four times. were seeded on a coverslip and 24 h later were fixed and subjected to IF, using the appropriate antibodies (Table S3).

RNA Preparation and Real-Time qPCR

Total RNA was isolated from cells subjected to one to two transfections with si-NT or si-hVDAC1, as indicated in the figures, using the RNeasy mini kit (QIAGEN) according to the manufacturer's instructions. Total RNA quality was analyzed, complementary DNA was synthesized, and real-time qPCR was performed with specific primers (Table S4) as described previously.[11] Copy numbers for each sample were calculated by the CT-based calibrated standard curve method. The results show fold changes (mean±SEM) of the three replicates.

Statistics

Means ± SEM of results obtained from independent experiments are presented. A difference was considered statistically significant when $^{*}p \leq 0.05$, $^{**}p \leq 0.01$, or $^{***}p \leq 0.001$.

SUPPLEMENTAL INFORMATION

Supplemental Information can be found online at https://doi.org/10.1016/j.omtn.2019.05.003.

AUTHOR CONTRIBUTIONS

T.A and Z.A. performed the research and analyzed the data, and V.S-B. assessed the results and wrote the paper.

CONFLICTS OF INTEREST

The authors declare no competing interests.

ACKNOWLEDGMENTS

This research was supported by the Phil and Sima Needleman Research Funds and Yafa and Ezra Yerucham Research Funds.

REFERENCES

1. Wenger, J.B., Chun, S.Y., Dang, D.T., Luesch, H., and Dang, L.H. (2011). Combination therapy targeting cancer metabolism. Med. Hypotheses *76*, 169–172.
2. Cairns, R.A., Harris, I.S., and Mak, T.W. (2011). Regulation of cancer cell metabolism. Nat. Rev. Cancer *11*, 85–95.
3. Turcotte, M.L., Parliament, M., Franko, A., and Allalunis-Turner, J. (2002). Variation in mitochondrial function in hypoxia-sensitive and hypoxia-tolerant human glioma cells. Br. J. Cancer *86*, 619–624.
4. Wolf, A., Agnihotri, S., and Guha, A. (2010). Targeting metabolic remodeling in glioblastoma multiforme. Oncotarget *1*, 552–562.
5. Majeed, R.H.A., Qurishi, Y., Qazi, A.K., Hussain, A., Ahmed, M., Najar, R.A., Bhat, J.A., Singh, S.K., and Saxena, A.K. (2012). Therapeutic Targeting of Cancer Cell Metabolism: Role of Metabolic Enzymes, Oncogenes and Tumor Suppressor Genes. J. Cancer Sci. Ther. *4*, 281–291.
6. Shoshan-Barmatz, V., De Pinto, V., Zweckstetter, M., Raviv, Z., Keinan, N., and Arbel, N. (2010). VDAC, a multi-functional mitochondrial protein regulating cell life and death. Mol. Aspects Med. *31*, 227–285.
7. Shoshan-Barmatz, V., Ben-Hail, D., Admoni, L., Krelin, Y., and Tripathi, S.S. (2015). The mitochondrial voltage-dependent anion channel 1 in tumor cells. Biochim. Biophys. Acta *1848*, 2547–2575.
8. Shoshan-Barmatz, V., Krelin, Y., Shteinfer-Kuzmine, A., and Arif, T. (2017). Voltage-Dependent Anion Channel 1 As an Emerging Drug Target for Novel Anti-Cancer Therapeutics. Front. Oncol. *7*, 154.
9. Arif, T., Vasilkovsky, L., Refaely, Y., Konson, A., and Shoshan-Barmatz, V. (2014). Silencing VDAC1 Expression by siRNA Inhibits Cancer Cell Proliferation and Tumor Growth In Vivo. Mol. Ther. Nucleic Acids *3*, e159.
10. Abu-Hamad, S., Sivan, S., and Shoshan-Barmatz, V. (2006). The expression level of the voltage-dependent anion channel controls life and death of the cell. Proc. Natl. Acad. Sci. USA *103*, 5787–5792.
11. Arif, T., Krelin, Y., Nakdimon, I., Benharroch, D., Paul, A., Dadon-Klein, D., and Shoshan-Barmatz, V. (2017). VDAC1 is a molecular target in glioblastoma, with its depletion leading to reprogrammed metabolism and reversed oncogenic properties. Neuro Oncol. *19*, 951–964.
12. Visvader, J.E., and Lindeman, G.J. (2012). Cancer stem cells: current status and evolving complexities. Cell Stem Cell *10*, 717–728.
13. Oskarsson, T., Batlle, E., and Massagué, J. (2014). Metastatic stem cells: sources, niches, and vital pathways. Cell Stem Cell *14*, 306–321.
14. Yuan, X., Curtin, J., Xiong, Y., Liu, G., Waschsmann-Hogiu, S., Farkas, D.L., Black, K.L., and Yu, J.S. (2004). Isolation of cancer stem cells from adult glioblastoma multiforme. Oncogene *23*, 9392–9400.
15. Rakha, E.A., El-Sayed, M.E., Green, A.R., Lee, A.H., Robertson, J.F., and Ellis, I.O. (2007). Prognostic markers in triple-negative breast cancer. Cancer *109*, 25–32.
16. Cleator, S., Heller, W., and Coombes, R.C. (2007). Triple-negative breast cancer: therapeutic options. Lancet Oncol. *8*, 235–244.
17. Ginestier, C., Hur, M.H., Charafe-Jauffret, E., Monville, F., Dutcher, J., Brown, M., Jacquemier, J., Viens, P., Kleer, C.G., Liu, S., et al. (2007). ALDH1 is a marker of normal and malignant human mammary stem cells and a predictor of poor clinical outcome. Cell Stem Cell *1*, 555–567.
18. Charafe-Jauffret, E., Ginestier, C., Bertucci, F., Cabaud, O., Wicinski, J., Finetti, P., Josselin, E., Adelaide, J., Nguyen, T.T., Monville, F., et al. (2013). ALDH1-positive cancer stem cells predict engraftment of primary breast tumors and are governed by a common stem cell program. Cancer Res. *73*, 7290–7300.
19. Charafe-Jauffret, E., Ginestier, C., Iovino, F., Tarpin, C., Diebel, M., Esterni, B., Houvenaeghel, G., Extra, J.M., Bertucci, F., Jacquemier, J., et al. (2010). Aldehyde dehydrogenase 1-positive cancer stem cells mediate metastasis and poor clinical outcome in inflammatory breast cancer. Clin. Cancer Res. *16*, 45–55.
20. Jiang, F., Qiu, Q., Khanna, A., Todd, N.W., Deepak, J., Xing, L., Wang, H., Liu, Z., Su, Y., Stass, S.A., and Katz, R.L. (2009). Aldehyde dehydrogenase 1 is a tumor stem cell-associated marker in lung cancer. Mol. Cancer Res. *7*, 330–338.
21. O'Flaherty, J.D., Barr, M., Fennell, D., Richard, D., Reynolds, J., O'Leary, J., and O'Byrne, K. (2012). The cancer stem-cell hypothesis: its emerging role in lung cancer biology and its relevance for future therapy. J. Thorac. Oncol. *7*, 1880–1890.
22. Eramo, A., Lotti, F., Sette, G., Pilozzi, E., Biffoni, M., Di Virgilio, A., Conticello, C., Ruco, L., Peschle, C., and De Maria, R. (2008). Identification and expansion of the tumorigenic lung cancer stem cell population. Cell Death Differ. *15*, 504–514.
23. Ho, M.M., Ng, A.V., Lam, S., and Hung, J.Y. (2007). Side population in human lung cancer cell lines and tumors is enriched with stem-like cancer cells. Cancer Res. *67*, 4827–4833.
24. Miyata, T., Yoshimatsu, T., So, T., Oyama, T., Uramoto, H., Osaki, T., Nakanishi, R., Tanaka, F., Nagaya, H., and Gotoh, A. (2015). Cancer stem cell markers in lung cancer. Personalized Medicine Universe *4*, 40–45.
25. Bonavia, R., Inda, M.M., Cavenee, W.K., and Furnari, F.B. (2011). Heterogeneity maintenance in glioblastoma: a social network. Cancer Res. *71*, 4055–4060.
26. TCGA Research Network (2013). The somatic genomic landscape of glioblastoma. Cell *155*, 462–477.
27. Network, T.C. (2013). Corrigendum: Comprehensive genomic characterization defines human glioblastoma genes and core pathways. Nature *494*, 506.
28. Parsons, D.W., Jones, S., Zhang, X., Lin, J.C., Leary, R.J., Angenendt, P., Mankoo, P., Carter, H., Siu, I.M., Gallia, G.L., et al. (2008). An integrated genomic analysis of human glioblastoma multiforme. Science *321*, 1807–1812.
29. Jeon, S.M., and Hay, N. (2015). The double-edged sword of AMPK signaling in cancer and its therapeutic implications. Arch. Pharm. Res. *38*, 346–357.
30. Sanli, T., Steinberg, G.R., Singh, G., and Tsakiridis, T. (2014). AMP-activated protein kinase (AMPK) beyond metabolism: a novel genomic stress sensor participating in the DNA damage response pathway. Cancer Biol. Ther. *15*, 156–169.
31. Li, W., Saud, S.M., Young, M.R., Chen, G., and Hua, B. (2015). Targeting AMPK for cancer prevention and treatment. Oncotarget *6*, 7365–7378.
32. Wullschleger, S., Loewith, R., and Hall, M.N. (2006). TOR signaling in growth and metabolism. Cell *124*, 471–484.
33. Guertin, D.A., and Sabatini, D.M. (2007). Defining the role of mTOR in cancer. Cancer Cell *12*, 9–22.
34. Galli, R., Binda, E., Orfanelli, U., Cipelletti, B., Gritti, A., De Vitis, S., Fiocco, R., Foroni, C., Dimeco, F., and Vescovi, A. (2004). Isolation and characterization of tumorigenic, stem-like neural precursors from human glioblastoma. Cancer Res. *64*, 7011–7021.
35. Singh, S.K., Hawkins, C., Clarke, I.D., Squire, J.A., Bayani, J., Hide, T., Henkelman, R.M., Cusimano, M.D., and Dirks, P.B. (2004). Identification of human brain tumour initiating cells. Nature *432*, 396–401.

36. Xavier, J.M., Morgado, A.L., Solá, S., and Rodrigues, C.M. (2014). Mitochondrial translocation of p53 modulates neuronal fate by preventing differentiation-induced mitochondrial stress. Antioxid. Redox Signal. *21*, 1009–1024.

37. Derouiche, A., and Frotscher, M. (2001). Peripheral astrocyte processes: monitoring by selective immunostaining for the actin-binding ERM proteins. Glia *36*, 330–341.

38. Cleary, M.A., Uboha, N., Picciotto, M.R., and Beech, R.D. (2006). Expression of ezrin in glial tubes in the adult subventricular zone and rostral migratory stream. Neuroscience *143*, 851–861.

39. Mishima, K., Kato, Y., Kaneko, M.K., Nishikawa, R., Hirose, T., and Matsutani, M. (2006). Increased expression of podoplanin in malignant astrocytic tumors as a novel molecular marker of malignant progression. Acta Neuropathol. *111*, 483–488.

40. Yeung, S.J., Pan, J., and Lee, M.H. (2008). Roles of p53, MYC and HIF-1 in regulating glycolysis – the seventh hallmark of cancer. Cell. Mol. Life Sci. *65*, 3981–3999.

41. Nur-E-Kamal, A., Gross, S.R., Pan, Z., Balklava, Z., Ma, J., and Liu, L.F. (2004). Nuclear translocation of cytochrome c during apoptosis. J. Biol. Chem. *279*, 24911–24914.

42. Zhao, S., Aviles, E.R., Jr., and Fujikawa, D.G. (2010). Nuclear translocation of mitochondrial cytochrome c, lysosomal cathepsins B and D, and three other death-promoting proteins within the first 60 minutes of generalized seizures. J. Neurosci. Res. *88*, 1727–1737.

43. Lin, V.C., Jin, R., Tan, P.H., Aw, S.E., Woon, C.T., and Bay, B.H. (2003). Progesterone induces cellular differentiation in MDA-MB-231 breast cancer cells transfected with progesterone receptor complementary DNA. Am. J. Pathol. *162*, 1781–1787.

44. Al-Hajj, M., Wicha, M.S., Benito-Hernandez, A., Morrison, S.J., and Clarke, M.F. (2003). Prospective identification of tumorigenic breast cancer cells. Proc. Natl. Acad. Sci. USA *100*, 3983–3988.

45. Mao, P., Wu, S., Li, J., Fu, W., He, W., Liu, X., Slutsky, A.S., Zhang, H., and Li, Y. (2015). Human alveolar epithelial type II cells in primary culture. Physiol. Rep. *3*, e12288.

46. Arif, T., Paul, A., Krelin, Y., Shteinfer-Kuzmine, A., and Shoshan-Barmatz, V. (2018). Mitochondrial VDAC1 silencing leads to metabolic rewiring and the reprograming of tumour cells to advanced differentiated states. Cancers (Basel) *10*, 499.

47. Carracedo, A., Cantley, L.C., and Pandolfi, P.P. (2013). Cancer metabolism: fatty acid oxidation in the limelight. Nat. Rev. Cancer *13*, 227–232.

48. Ríos, M., Foretz, M., Viollet, B., Prieto, A., Fraga, M., Costoya, J.A., and Señarís, R. (2013). AMPK activation by oncogenesis is required to maintain cancer cell proliferation in astrocytic tumors. Cancer Res. *73*, 2628–2638.

49. Eichner, L.J., Brun, S.N., Herzig, S., Young, N.P., Curtis, S.D., Shackelford, D.B., Shokhirev, M.N., Leblanc, M., Vera, L.I., Hutchins, A., et al. (2019). Genetic Analysis Reveals AMPK Is Required to Support Tumor Growth in Murine Kras-Dependent Lung Cancer Models. Cell Metab. *29*, 285–302.e7.

50. Meyuhas, O. (2015). Ribosomal Protein S6 Phosphorylation: Four Decades of Research. Int. Rev. Cell Mol. Biol. *320*, 41–73.

51. Yoshida, G.J., and Saya, H. (2016). Therapeutic strategies targeting cancer stem cells. Cancer Sci. *107*, 5–11.

52. Islam, F., Qiao, B., Smith, R.A., Gopalan, V., and Lam, A.K. (2015). Cancer stem cell: fundamental experimental pathological concepts and updates. Exp. Mol. Pathol. *98*, 184–191.

53. Plaks, V., Kong, N., and Werb, Z. (2015). The cancer stem cell niche: how essential is the niche in regulating stemness of tumor cells? Cell Stem Cell *16*, 225–238.

54. Su, Z., Zang, T., Liu, M.L., Wang, L.L., Niu, W., and Zhang, C.L. (2014). Reprogramming the fate of human glioma cells to impede brain tumor development. Cell Death Dis. *5*, e1463.

55. Park, S.Y., Lee, H.E., Li, H., Shipitsin, M., Gelman, R., and Polyak, K. (2010). Heterogeneity for stem cell-related markers according to tumor subtype and histologic stage in breast cancer. Clin. Cancer Res. *16*, 876–887.

56. Ricardo, S., Vieira, A.F., Gerhard, R., Leitão, D., Pinto, R., Cameselle-Teijeiro, J.F., Milanezi, F., Schmitt, F., and Paredes, J. (2011). Breast cancer stem cell markers CD44, CD24 and ALDH1: expression distribution within intrinsic molecular subtype. J. Clin. Pathol. *64*, 937–946.

57. Ugorski, M., Dziegiel, P., and Suchanski, J. (2016). Podoplanin - a small glycoprotein with many faces. Am. J. Cancer Res. *6*, 370–386.

58. Astarita, J.L., Acton, S.E., and Turley, S.J. (2012). Podoplanin: emerging functions in development, the immune system, and cancer. Front. Immunol. *3*, 283.

59. Martín-Villar, E., Borda-d'Agua, B., Carrasco-Ramirez, P., Renart, J., Parsons, M., Quintanilla, M., and Jones, G.E. (2015). Podoplanin mediates ECM degradation by squamous carcinoma cells through control of invadopodia stability. Oncogene *34*, 4531–4544.

60. Chuang, W.Y., Yeh, C.J., Chao, Y.K., Liu, Y.H., Chang, Y.S., Tseng, C.K., Chang, H.K., Wan, Y.L., and Hsueh, C. (2014). Concordant podoplanin expression in cancer-associated fibroblasts and tumor cells is an adverse prognostic factor in esophageal squamous cell carcinoma. Int. J. Clin. Exp. Pathol. *7*, 4847–4856.

61. Neri, S., Ishii, G., Hashimoto, H., Kuwata, T., Nagai, K., Date, H., and Ochiai, A. (2015). Podoplanin-expressing cancer-associated fibroblasts lead and enhance the local invasion of cancer cells in lung adenocarcinoma. Int. J. Cancer *137*, 784–796.

62. Shen, L., Sun, X., Fu, Z., Yang, G., Li, J., and Yao, L. (2012). The fundamental role of the p53 pathway in tumor metabolism and its implication in tumor therapy. Clin. Cancer Res. *18*, 1561–1567.

63. Berkers, C.R., Maddocks, O.D., Cheung, E.C., Mor, I., and Vousden, K.H. (2013). Metabolic regulation by p53 family members. Cell Metab. *18*, 617–633.

64. Lin, T., Chao, C., Saito, S., Mazur, S.J., Murphy, M.E., Appella, E., and Xu, Y. (2005). p53 induces differentiation of mouse embryonic stem cells by suppressing Nanog expression. Nat. Cell Biol. *7*, 165–171.

65. Tang, C.M., and Yu, J. (2013). Hypoxia-inducible factor-1 as a therapeutic target in cancer. J. Gastroenterol. Hepatol. *28*, 401–405.

66. Mimeault, M., and Batra, S.K. (2013). Hypoxia-inducing factors as master regulators of stemness properties and altered metabolism of cancer- and metastasis-initiating cells. J. Cell. Mol. Med. *17*, 30–54.

67. Chen, J.Q., and Russo, J. (2012). Dysregulation of glucose transport, glycolysis, TCA cycle and glutaminolysis by oncogenes and tumor suppressors in cancer cells. Biochim. Biophys. Acta *1826*, 370–384.

68. Wang, R., Dillon, C.P., Shi, L.Z., Milasta, S., Carter, R., Finkelstein, D., McCormick, L.L., Fitzgerald, P., Chi, H., Munger, J., and Green, D.R. (2011). The transcription factor Myc controls metabolic reprogramming upon T lymphocyte activation. Immunity *35*, 871–882.

OMTN, Volume *17*

Supplemental Information

Metabolic Reprograming Via Silencing of Mitochondrial VDAC1 Expression Encourages Differentiation of Cancer Cells

Tasleem Arif, Zohar Amsalem, and Varda Shoshan-Barmatz

Supplementary Data

Metabolic Reprograming via Silencing of Mitochondrial VDAC1 Expression Encourages Differentiation of Cancer Cells

Tasleem Arif,* Zohar Amsalem* and Varda Shoshan-Barmatz[#]

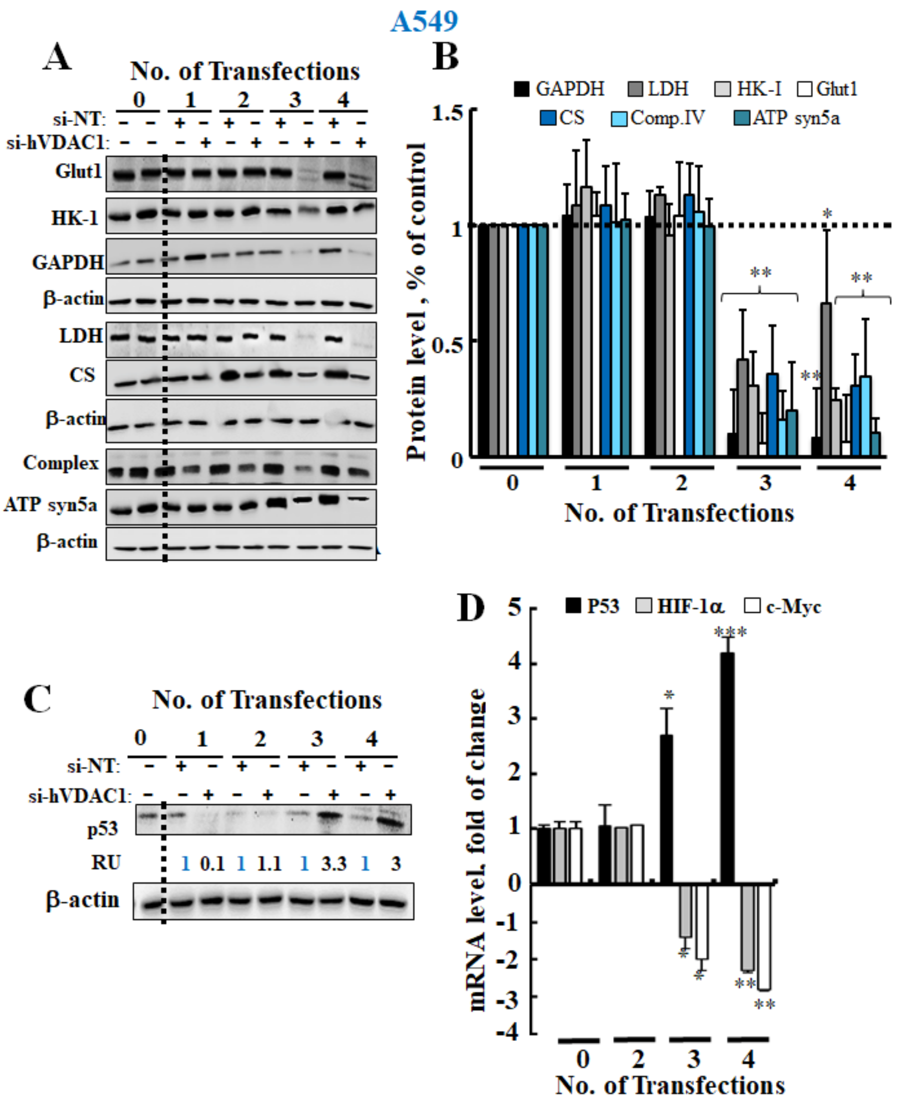

Figure S1. si-hVDAC1 Treatment of A549 Cells Reprogrammed Metabolism and Altered TF Expression Following the Third and Fourth Transfections

Immunoblotting (**A**) and quantitative analysis (**B**) of Glut-1, HK-I, GAPDH, LDH-A, CS, complex IVc and ATP 5a synthase in si-NT- or si-hVDAC1-treated A549 cells following the indicted transfection. β-actin served as an internal loading control. Results reflect the mean ± SEM. The dashed line indicates the level in the controls. **(C)** Immunoblot analysis of p53 levels in si-NT- and si-hVDAC1-treated A549 cells following the indicated transfection. RU represents the relative units (RU) of expression of the indicated protein in si-hVDAC1-treated A549 cells relative to si-NT-treated A549 cells. **(D)** TF mRNA expression levels as analyzed using qRT-PCR with specific primers. The levels of p53 (black bar), HIF-1α (gray bar) and c-Myc (white bar) in si-hVDAC1-treated A549 cells relative to those in si-NT-treated A549 cells following the indicated transfection were determined. Results reflect the mean ± SEM, $p^* \leq 0.05$; $p^{**} \leq 0.01$; $p^{***} \leq 0.001$.

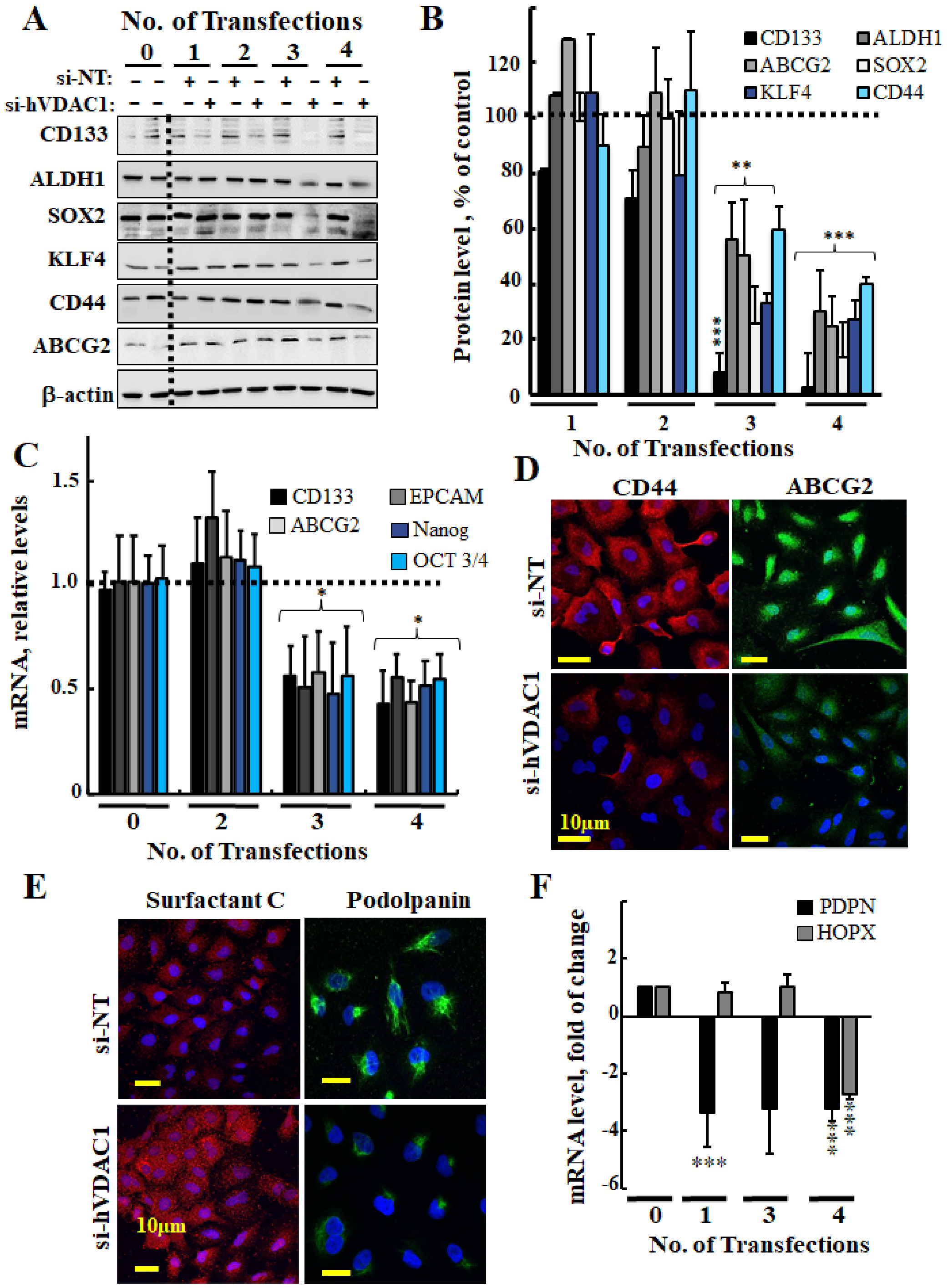

Figure S2. si-hVDAC1 Treatment of A549 Cells Markedly Reduced the Expression of CSC Markers and Proteins Associated with Differentiation Following the Third and Fourth Transfections

Immunoblotting (**A**) and quantitative analysis (**B**) of selected CSC markers (CD133, ALDH1, SOX2, KLF4, CD44 and ABCG2) in si-NT- and si-hVDAC1-treated A549 cells at the indicated transfection, using specific antibodies. β-actin served as an internal loading control. The dashed line indicates the level in the controls. (**C**) q-RT-PCR of mRNA levels of CSC markers (CD133, EPCAM, ABCG2, Nango and Oct3/4) in A549 cells treated with si-NT or si-hVDAC1 for the indicated number of transfections, and presented relative to their levels in si-NT-treated cells. Results reflect the mean ± SEM, p: *≤0.05. **(D,E)** IF staining of si-NT- and si-hVDAC-treated cells following the fourth transfection stained for CD44 and ABCG2 (**D**) and surfactant C, and for podoplanin after third transfection (**E**), as visualized by confocal microscopy. **(F)** qRT-PCR analysis of mRNA levels of podoplanin (PDPN, black bar), and homedomain only protein x (HOPX, gray bar) in si-hVDAC1-treated A549 cells, relative to those in si-NT-treated A549 cells, following the indicated transfection. Results reflect the means ± SEM; p**≤0.01; p***≤0.001.

Table S1. Summary of the Genes Expression Levels Associated with Metabolism, Stem Cells, Differentiation and Transcription Factors as a Function of Number of Transfections of MDA-MB231 Cells with si-hVDAC1, Relative to Transfection with si-NT; q-PCR Results

	**Fold change si-NT/si-hVDAC1**			
**Transfection No**	**0**	**2**	**3**	**4**
**Gene**				
**Metabolism**				
Glut1	1.00 ± 0.15	-0.56 ± 0.61	-1.05 ± 0.51	-2.74 ±0.34
HK-1	1.00 ± 0.36	-1.07 ± 0.06	-3.84 ± 0.14	-9.86 ± 0.15
GAPDH	1.00 ± 0.08	-1.83 ± 0.29	-3.66 ± 0.26	-10.59 ± 0.05
LDH	1.00 ± 0.07	-0.67 ± 0.10	-1.34 ± 0.08	-2.72 ± 0.22
CS	1.00 ± 0.22	-0.78 ± 0.13	-1.01 ± 0.33	-2.56 ± 0.35
ATP syn5a	1.00 ± 0.10	-1.4 ± 0.09	-1.81 ± 0.0.4	-6.35 ± 0.04
VDAC1	1.00 ± 0.00	-3.49 ± 0.15	-4.72 ± 0.08	-4.76 ± 0.07
VDAC2	1.00 ± 0.10	-1.15 ± 0.29	-1.04 ± 0.26	-0.81 ± 0.05
VDAC3	1.00 ± 0.00	-0.94 ± 0.33	-0.913 ± 0.14	-0.90 ± 0.15
**Differentiation**				
PR	1.00	1.05±0.24	1.51±0.41	3.051±0.70
PRL	1.00	1.94±0.35	2.55±0.21	1.95±0.17
ESR1	1.00	7.98±1.68	7.61±1.37	5.34±1.73
**TF**				
p53	1.00 ± 0.03	1.12 ± 0.01	6.29 ± 0.38	6.00 ± 0.36
c-MYC	1.00 ± 0.05	-1.06 ± 0.08	-3.13 ± 0.14	-2.64 ± 0.04
HIF1-alpha	1.04 ± 0.05	-1.06 ± 0.09	-1.25 ± 0.05	-4.51± 0.04

Table S2. Summary of The Expression Levels of Genes Associated with Metabolism, Stem Cells, Differentiation and Transcription Factors as a Function of The Number of Transfections of A549 Cells with si-hVDAC1 Relative to Transfection with si-NT; q-PCR Results

	**Fold change si-NT/siVDAC1**			
**Transfection No:**	**0**	**2**	**3**	**4**
**Gene**				
**Metabolism**				
Glut1	1.00 ± 0.1	-1.37 ± 0.12	-2.60 ± 0.21	-3.09 ± 0.45
HK-1	1.00 ± 0.01	-2.37 ± 0.23	-1.03 ± 0.09	-13.09 ± 1.97
LDH	1.00 ± 0.07	-0.67 ± 0.10	-1.34 ± 0.08	-2.72 ± 0.22
CS	1.00 ± 0.13	-0.93 ± 0.08	-1.9 ± 0.21	-4.12 ± 0.67
ATP syn5a	1.00 ± 0.01	-1.00 ± 0.10	-2.02 ± 0.26	-3.99 ± 0.54
VDAC1	1.00 ± 0.05	-2.84 ± 0.05	-8.46 ± 0.05	-6.76 ± 0.01
VDAC2	1.00 ± 0.02	-1.19 ± 0.01	-1.17 ± 0.10	-1.20 ± 0.05
VDAC3	1.00 ± 0.24	-1.11 ± 0.30	- 0.96 ± 0.14	-0.74 ± 0.05
**Stem Cell**				
CD133	1.00 ± 0.02	-1.13 ± 0.22	-1.71 ± 0.13	-2.24 ± 0.15
EPCAM	1.00 ± 0.05	-0.76 ± 0.15	-2.00 ± 0.15	-1.89 ± 0.12
ABCG2	1.00 ± 0.09	-0.98 ± 0.22	-1.74 ± 0.13	-2.31 ± 0.15
NANOG	1.00 ± 0.01	-0.89 ± 0.20	-2.12 ± 0.25	-1.99 ± 0.24
OCT3/4	1.00 ± 0.05	-0.95 ± 0.10	-1.83 ± 0.11	-1.88 ± 0.11
**Differentiation**				
PDPN	1.00 ± 0.01	0.84 ± 0.32	1.02 ± 0.42	-2.71 ± 0.16
HOPX	1.00 ± 0.01	-3.36 ±1.63	-3.23 ± 1.58	-3.24 ± 0.40
**TF**				
p53	1.00 ± 0.05	1.05 ± 0.20	2.69 ± 0.05	4.19 ± 0.05
c-MYC	1.00 ± 0.05	-1.02 ± 0.24	-1.96 ± 0.30	-2.84 ± 0.05
HIF1-alpha	1.04 ± 0.05	-1.06 ± 0.50	-1.40 ± 0.30	-2.37 ± 0.05

Table S3. Antibodies Used in This Study

Antibodies against the indicated protein, their catalogue number, source, and the dilutions used in immunoblot and immunofluorescence experiments are presented.

Antibodies	Source and Cat. No.	Dilution		
		IHC	WB	IF
Mouse monoclonal anti-β-actin	Millipore, Billerica, MA, MAB1501	-	1:10000	-
Rabbit monoclonal anti-VDAC1	Abcam, Cambridge, UK , ab154856	-	1:5000	-
Rabbit monoclonal cytochrome c oxidase subunit IVc	Abcam, Cambridge, UK, ab150422	-	1:2000	-
Rabbit polyclonal anti -NGFR	Santa Cruz Biotechnology, Inc. Dallas, TX, sc-8317	-	1:1000	-
Rabbit monoclonal anti-ALDH1	Abcam, Cambridge, UK , ab52492	-	1:2000	-
Rabbit polyclonal anti-BCL-xL	Abcam, Cambridge, UK , ab98143	-	1:4000	-
Mouse monoclonal anti-Ezrin	Santa Cruz Biotechnology, Inc. Dallas, TX, sc-58758	-	-	1:500
Mouse monoclonal anti-Podoplanin	Abcam, Cambridge, UK, ab10288	-	-	1:500
Rabbit monoclonal anti-Glut1	Abcam, Cambridge, UK ab40084	-	1: 1500	-
Mouse monoclonal anti-GAPDH	Abcam, Cambridge, UK, ab9484	-	1:1000	-
Rabbit polyclonal anti-citrate synthase	Abcam, Cambridge, UK ab96600	-	1:4000	-
Mouse monoclonal anti-Cytochrome *c*	B.D Bioscience, San Jose, CA, Cat. No 556432	-	1:2000	-
Mouse monoclonal anti-ATP5a	Abcam, Cambridge, UK, ab14748	-	1:1000	-
Mouse monoclonal anti-HK-I	Abcam, Cambridge, UK ab105213	-	1:2000	-
Goat polyclonal anti-LDH-A	Santa Cruz Biotechnology, Inc. Dallas, TX, sc-27230	-	1:1500	-
Rabbit monoclonal anti-LDH	Epitomics, Cambridge, UK, 1980-1	-	1:1000	-
Rabbit polyclonal anti-Nestin	Millipore, Billerica, MA, MAB353	-	1:1000	1:500
Goat polyclonal anti-Sox2	Santa Cruz Biotechnology, Inc. Dallas, TX, sc-17320	-	1:1500	1:300
Rabbit polyclonal anti-KLF4	IMGENX Littleton, USA, IMG-6081-A	-	1:1000	-
Mouse monoclonal anti-CD133	Miltenyi Biotec GmbH , AC133	-	1:1500	-
Rabbit polyclonal anti-Prosurfactant protein C	Abcam, ab90716	-	-	1:250
Mouse monoclonal anti-β III tubulin	Abcam, Cambridge, UK ab7751	-	1:5000	1:200
Mouse monoclonal anti-GFAP	Santa Cruz Biotechnology, Inc. Dallas, TX, sc-33673	-	1:5000	1:150
Rabbit polyclonal anti-AMPK-p	Abcam, Cambridge, UK, ab23875	-	1:5000	-
Rabbit polyclonal anti-pS6	Cell Signaling Technology, Danvers, USA, cat: 2215	-	1:1000	-
Mouse monoclonal anti-P53	Santa Cruz Biotechnology, Inc. ,Dallas, TX, sc-126	-	1:5000	-

Mouse monoclonal anti-ABCG2	GeneTex, Irvine, CA, cat: GTX60447	-	1:2000	1:500
Rabbit polyclonal anti-CD44	Abcam, Cambridge, UK, ab157107	-	1:3000	1:1000
Mouse monoclonal anti-ERB2/HER-2	Biolegend, San Diego, CA, cat: 324401	-	-	1:500
Alexa Fluor 488 anti-mouse/human CD44	Biolegend, San Diego, CA, cat: 103015	-	-	1:500
Goat anti-Rabbit IgG H&L (Alexa Fluor 555)	Abcam, Cambridge, UK, ab150086	-	-	1:500
Cow polyclonal anti-goat IgG FITC	Santa Cruz Biotechnology, Inc. Dallas, TX, sc-2348	-	-	1:500
Mouse monoclonal anti-CD24	Biolegend, San Diego, CA, cat: 311101	-	-	1:500

Table S4: Real-Time PCR Primers Used in This Study

The genes examined and the forward and reverse sequences of the primers used are indicated.

**Gene**	**Primer sequences**
ß-Actin	Forward 5’-ACTCTTCCAGCCTTCCTTCC-3’<br>Reverse 5’- TGTTGGCGTACAGGTCTTTG-3’
VDAC1	Forward 5’- AATGACGGGACAGAGTTTGG<br>Reverse 5’- AGCGCGTGTTACTGTTTCCT
VDAC2	Forward 5’- GGGGACTTCCAGCTACACAC-3’<br>Reverse 5’- CCTGATGTCCAAGCAAGGTT-3’
VDAC3	Forward 5’- AATAATGCCAGCCTGATTGG-3’<br>Reverse 5’- CTTGTGACCTCCTGCACTGA-3’
GLUT1	Forward 5’- GGCCATCTTTTCTGTTGGGG-3’<br>Reverse 5’- TCAGCATTGAATTCCGCCG-3’
GAPDH	Forward 5’- TGGAAGGACTCATGACCACA-3’<br>Reverse 5’- ATGATGTTCTGGAGAGCCCC-3’
LDH-A	Forward 5’-GCAGGTGGTTGAGAGTGCTT-3’<br>Reverse 5’- GCACCCGCCTAAGATTCTTC-3’
CS	Forward 5’- AGGAACAGGTATCTTGGCTCT-3’<br>Reverse 5’- GGGGTGTAGATTGGTGGGAA-3’
ATP syn5a	Forward 5’-TCAGTCTACGCCGCACTTAC-3’<br>Reverse 5’- GACATCTCAGCAGTCCCACA-3’
HK-I	Forward 5’-GTCTCAGTCCAGCACGTTTG-3’<br>Reverse 5’- GAAACGCCGGGAATACTGTG-3’
KLF4	Forward 5’-TGCCCCGAATAACCGCTG-3’<br>Reverse 5’-CGTTGAACTCCTCGGTCTCT-3’
ABCG2	Forward 5’- CACCTTATTGGCCTCAGGAA-3’<br>Reverse 5’- GAAACACTGGTTGGTCGTCA -3’
CD133	Forward 5′-TGGGCTTGTCATAACAGGAT-3′<br>Reverse 5′-TTGCGGTAAAACTGGCTAAG-3′
EPCAM	Forward 5′-CTGGCCGTAAACTGCTTTGT-3′<br>Reverse 5′-TCCCAAGTTTTGAGCCATTC-3′
Nestin	Forward 5’- GAAACAGCCATAGAGGGCAAA-3’<br>Reverse 5’- TGGTTTTCCAGAGTCTTCAGTGA-3’

Nanog	Forward 5’- TGGGATTTACAGGCGTGAGCCAC-3’<br>Reverse 5’- AAGCAAAGCCTCCCAATCCCAAAC-3’
SOX2	Forward 5’- CCATGCAGGTTGACACCGTTG -3’<br>Reverse 5’-TCGGCAGACTGATTCAAATAA-3’
TUBB3	Forward 5’-CTCAGGGGCCTTTGGACATC-3’<br>Reverse 5’-CAGGCAGTCGCAGTTTTCAC-3’
MAP2	Forward 5’-TCCAAAATCGGATCAACAGAC-3’<br>Reverse 5’- AGAGCCACATTTGGATGTCAC-3’
GFAP	Forward 5’- AAGCTCCAGGATGAAACCAAC-3’<br>Reverse 5’- AGCGACTCAATCTTCCTCTCC-3’
c-Myc	Forward 5’-GTAGTGGAAAACCAGCAGCC-3’<br>Reverse 5’- CCTCCTCGTCGCAGTAGAAA-3’
p53	Forward 5′-AGGTTGGCTCTGACTGTACC-3′<br>Reverse 5′-AAAGCTGTTCCGTCCCAGTA-3
Hif-1 α	Forward 5’-CTGACCCTGCACTCAATCAA-3’<br>Reverse 5’- TCCATCGGAAGGACTAGGTG-3’
Cytochrome c	Forward 5′-TTTGGATCCAATGGGTGATGTTGAG-3<br>Reverse 5'TTGAATTCCTCATTAGTAGCTTTTTTGAG-3
HOPX	Forward 5’- TTAAGCAGGCTGGCATCAG-3’<br>Reverse 5’- TGCTCCGCTAGACCCTTCT-3’
Podoplanin	Forward 5’- ATCTGCCAACTTCAGAAAGCA-3’<br>Reverse 5’- TTGTCTGTGTGTCTCCATCCA-3’
PRLR	Forward 5’-AATCTTGGCAGAGGCAGAAA-3’<br>Reverse 5’-TTTGGAGCTATTCCCATTGC-3’
PR	Forward 5’- GTCTACCCGCCCTATCTCAAC-3’<br>Reverse 5’- ACCATAATGACAGCCTGATGC-3’
ESRI	Forward 5’-TGGAGATCTTCGACATGCTG -3’<br>Reverse 5’- TCCAGAGACTTCAGGGTGCT -3’
